# Chemokine binding protein ‘M3’ limits atherosclerosis in apolipoprotein E^-/-^ mice

**DOI:** 10.1371/journal.pone.0173224

**Published:** 2017-03-10

**Authors:** Dhanya Ravindran, Anisyah Ridiandries, Laura Z. Vanags, Rodney Henriquez, Siân Cartland, Joanne T. M. Tan, Christina A. Bursill

**Affiliations:** 1 Heart Research Institute, Newtown, Sydney, New South Wales, Australia; 2 Sydney Medical School, University of Sydney, Camperdown, Sydney, New South Wales, Australia; "INSERM", FRANCE

## Abstract

Chemokines are important in macrophage recruitment and the progression of atherosclerosis. The ‘M3’ chemokine binding protein inactivates key chemokines involved in atherosclerosis (e.g. CCL2, CCL5 and CX_3_CL1). We aimed to determine the effect of M3 on plaque development and composition. *In vitro* chemotaxis studies confirmed that M3 protein inhibited the activity of chemokines CCL2, CCL5 and CX_3_CL1 as primary human monocyte migration as well as CCR2-, CCR5- and CX_3_CR1-directed migration was attenuated by M3. *In vivo*, adenoviruses encoding M3 (AdM3) or green fluorescence protein (AdGFP; control) were infused systemically into apolipoprotein (apo)-E^-/-^ mice. Two models of atherosclerosis development were used in which the rate of plaque progression was varied by diet including: (1) a ‘rapid promotion’ model (6-week high-fat-fed) and (2) a ‘slow progression’ model (12-week chow-fed). Plasma chemokine activity was suppressed in AdM3-infused mice as indicated by significantly less monocyte migration towards AdM3 mouse plasma *ex vivo* (29.56%, *p* = 0.014). In the ‘slow progression’ model AdM3 mice had reduced lesion area (45.3%, *p* = 0.035) and increased aortic smooth muscle cell α-actin expression (60.3%, *p* = 0.014). The reduction in lesion size could not be explained by changes in circulating inflammatory monocytes as they were higher in the AdM3 group. In the ‘rapid promotion’ model AdM3 mice had no changes in plaque size but reduced plaque macrophage content (46.8%, *p* = 0.006) and suppressed lipid deposition in thoracic aortas (66.9%, *p*<0.05). There was also a reduction in phosphorylated p65, the active subunit of NF-κb, in the aortas of AdM3 mice (37.3%, *p*<0.0001). M3 inhibited liver CCL2 concentrations in both models with no change in CCL5 or systemic chemokine levels. These findings show M3 causes varying effects on atherosclerosis progression and plaque composition depending on the rate of lesion progression. Overall, our studies support a promising role for chemokine inhibition with M3 for the treatment of atherosclerosis.

## Introduction

Atherosclerosis is a chronic inflammatory disease in which monocyte recruitment plays an important role in disease initiation, plaque progression, and clinical events such as chronic ischemia, plaque rupture and thrombosis [1]. Chemokines are chemoattractant cytokines that direct the migration of specific leukocytes to sites of inflammation or infection and are increasingly implicated in atherosclerosis [2]. Chemokines are small 8- to 11-kDa proteins that are divided into four structural subfamilies (C, CC, CXC and CX_3_C) based on the placement and number of cysteine residue at the N terminal [3–5]. The ability of chemokines to bind to multiple receptors and also for receptors to bind to multiple chemokines indicate some redundancy in chemokine signalling. However, chemokine/chemokine receptors interactions are specific within each chemokine group [6].

To date, the CC-chemokine family has been strongly implicated in atherosclerosis with a host of CC-chemokines including MCP-1 (CCL2), RANTES (CCL5) and MIP-1α (CCL3) detected in human atherosclerotic lesions [7]. The importance of CC-chemokines in atherosclerosis has been reported in studies using transgenic or knockout murine models. For example, targeted deletions of the CCL2 gene or its receptor (CCR2) resulted in reduced atherosclerotic lesion formation [8]. Furthermore, studies using an amino-terminal deletion mutant of CCL2 [9] and a modified CCL5 peptide (Met-RANTES/CCL5) [10] found reductions in atherosclerotic lesion development in apoE^-/-^ mice. Broad-spectrum inhibition of the CC-chemokine class with the vaccinia viral protein ‘35K’ was also found to inhibit macrophage recruitment, lipid deposition and atherosclerotic plaque size [11]. More recently the importance of CX_3_CL1 and its receptor (CX_3_CR1) in atherosclerosis has emerged. CX_3_CL1 has been detected in macrophages, foam cells and medial smooth muscle cells (SMC) of atherosclerotic human coronary arteries [12]. Furthermore, apoE^-/-^/CX_3_CR1^-/-^ mice have significantly smaller atherosclerotic lesions [13, 14]. These studies provide concrete evidence that chemokines from the CC and the CX_3_C subclasses play a key role in atherosclerosis. This raises the possibility that broad-spectrum chemokine inhibition across the CC and CX_3_C chemokine subfamilies would be very effective in preventing atherosclerotic disease progression. M3 is a 44-kDa protein encoded by the murine gamma herpesvirus 68 (MHV-68) that serves to evade the host immune response by binding and inactivating chemokines [15]. Unlike CC-chemokine inhibitor 35K, the M3 protein is capable of sequestering members from all chemokine classes, specifically key chemokines involved in atherosclerosis progression including CCL2, CCL5 and CX_3_CL1, while displaying selectivity in its binding to individual members [16]. M3 may have improved efficacy over single chemokine inhibition or the targeting of a single chemokine class due to redundancies in chemokine signaling.

We therefore sought to evaluate the effect of broad-spectrum chemokine inhibition using the M3 protein on atherosclerosis in the apolipoprotein (apo) E^-/-^ mouse model. We found that M3 suppressed chemokine activity *in vitro* and *in vivo*. In two different models of atherosclerosis development in which the rate of lesion progression was varied by diet (high-fat or chow), overexpression of M3 in apoE^-/-^ mice caused reductions in plaque size and macrophage content. These changes differed between the two models indicating that the effects of M3 were dependent on the rate of plaque progression. Chemokine blockade with M3 may be a promising strategy for the treatment of atherosclerosis, although some consideration needs to be given to the aggressiveness of the onset.

## Materials and methods

### Generation of M3 adenovirus

A recombinant adenovirus M3 (AdM3) was generated as described previously [17]. Briefly, the M3 open reading frame was cloned into a pshuttle CMV (ps) plasmid along with a myc-His epitope tag and homologously recombined with the AdEasy viral plasmid to create ‘AdEasyM3’. Linearised AdEasyM3 was then transfected into Ad293 cells (293AD cell line, AD-100, Cell Biolabs Inc, USA) to create recombinant AdM3 virus. Purified adenovirus particles (vp) were isolated from the cell pellet of AdM3 infected Ad293 cells by CsCl gradient ultracentrifugation and desalted using CL-4B sepharose columns (Sigma-Aldrich, USA). Purified virus was diluted 1:1 with a mix of 80% fetal bovine serum (FBS) and 20% glycerol and aliquots of 1x10^11^ vp were frozen at -80°C for *in vivo* experimentation. A control recombinant adenovirus encoding enhanced green fluorescent protein (AdGFP) was prepared as described above. Anti-c-Myc tagged agarose-conjugated beads (Sigma-Aldrich, USA) were used to isolate soluble c-Myc-tagged M3 from AdM3 viral media. The media was run through anti-c-Myc agarose in a column and eluted with 0.1 M ammonium hydroxide into 1 N acetic acid to neutralize and snap frozen at -80°C.

### Isolation of human monocytes

White-cell concentrates were obtained from the peripheral blood of healthy human volunteers (Red Cross Blood Bank), and monocytes were removed within 24 h of collection by density gradient separation of the white blood cells on Lymphoprep (Axis-Shield, UK) followed by counterflow centrifugation elutriation using a Beckman Avanti J-26 XPI centrifuge equipped with a JE-5.0 elutriation rotor and a 4.0 mL elutriation chamber (Beckman Instruments Inc., USA) at 21°C, as described previously [18]. Collected fractions were examined by a Cytospin system (Shandon, USA) and Wrights’ stain (DiffQuik; Laboratory-Aids, Australia). Monocyte purity of >90% and viability of >95% by Trypan Blue exclusion were confirmed by light microscopy, and the monocytes were resuspended in serum-free RPMI and used immediately for chemotaxis studies.

### *In vitro* testing of chemokine activity using chemotaxis assays

CCR2-, CCR5- and CX_3_CR1-directed cell migration was assessed in 8 μm pore size transwell membranes (ChemoTX, 6.0 mm diameter, 8 μm pore size, Receptor Technologies, UK). 293T cells were co-transfected (Fugene^®^6, Roche Diagnostics, Germany) with plasmids encoding CCR2, CCR5 or CX_3_CR1 plus GFP to facilitate visualization. Transfected cells (1x10^6^/membrane) were harvested and allowed to migrate overnight toward purified recombinant CCL2, CCL5 or CX_3_CL1 (Research Diagnostics Inc, USA) in the presence of increasing concentrations of M3 protein (0–500 ng/mL) placed in the lower chamber. Migrated cells were fixed and quantified by computer analysis of GFP fluorescence (green cell pixel count) in microscope images using Image-Pro^®^ software (v9.0.4, Media Cybernetics, USA). Each experimental sample was analyzed in triplicate, and 3 separate images quantified per membrane. Cell migration of human elutriated monocytes in response to recombinant chemokines was also tested *in vitro* using the Boyden chamber method as described earlier. To the bottom chambers, purified M3 protein (100ng/ mL) was added to chemotaxis media with recombinant key inflammatory chemokines CCL2, CCL5, CX_3_CL1 as well as CXCL12, a chemoattractant that is not inhibited by M3 protein. To the upper chamber, 5x10^4^ cells/100 μL of Calcein AM (5nM)-labelled monocyte suspension was placed in each well and allowed to migrate towards the lower chamber for 1 h. Migrated cells were fixed in mounting medium with DAPI to counterstain for nuclei, and quantified by computer analysis of GFP fluorescence (green cell pixel count) relative to DAPI fluorescence (blue cell pixel count) in microscope images using Image-Pro^®^ software (v9.0.4, Media Cybernetics, USA). Each experimental sample was analyzed in triplicate, and 3 separate images quantified per membrane.

### Animals and gene transfer

All experimental procedures and protocols were conducted with approval from the Sydney Local Health District Animal Welfare Committee (Protocol Number: 2011/018) and conformed to the Guide for the Care and Use of Laboratory Animals (United States National Institute of Health). Approval was granted for the *in vivo* use of adenoviruses from the Royal Prince Alfred Hospital Institutional Biosafety Committee (IBC Code: 14–031). All procedures were performed under methoxyflurane anesthesia, and all efforts were made to minimize suffering. ApoE^-/-^ mice were used for this study to assess the effectiveness of M3 protein in two models that were subjected to different rates in the development of atherosclerosis by varying the diet. Model 1—a high fat diet (HFD)-fed model for more aggressive, rapid promotion of atherosclerosis over a 6-week period (‘rapid promotion’) and; Model 2—a chow-fed model for less aggressive progression of atherosclerosis over a 12-week period (‘slow progression’). For the rapid promotion model, 4-week-old mice were fed a HFD (22% milk fat, 0.15% cholesterol; SF00-219, Specialty Feeds, Australia) for 6 weeks in total (*n* = 10–12/group). Two weeks after commencement of the HFD AdM3 or AdGFP (1x10^11^ vp) were administered by tail-vein injection and mice continued on the HFD for a further 4 weeks. For the slow progression model, AdM3 or AdGFP (1x10^11^ vp) were administered intravenously to 8-week-old apoE^-/-^ mice. Mice were fed on a normal chow diet (Specialty Feeds, Australia), for 12 weeks post-adenoviral injection (*n* = 10–12/group). The stage of atherosclerosis at which M3 gene transfer was initiated and ended was estimated to be similar between the two models.

Following sacrifice, hearts were fixed overnight in 4% paraformaldehyde in phosphate buffered saline (PBS) then transferred to 70% ethanol the following day before embedding in paraffin. Descending thoracic aortas were thoroughly cleared of surrounding fat and connective tissue and stored in 70% ethanol at 4°C. Plasma was isolated from whole blood by centrifugation, aliquoted and frozen at -80°C. The livers, kidneys, spleens, lungs and aortic arches were also excised and immediately frozen at -80°C.

### Plasma lipids

Total cholesterol concentration was determined enzymatically on mouse plasma using the Cholesterol E kit (Wako Diagnostics, USA). HDL cholesterol concentration was determined by enzymatic assay following polyethylene glycol precipitation of apolipoprotein B containing lipoproteins. LDL levels were determined by subtracting total HDL from total cholesterol [19].

### Viral DNA expression

Genomic DNA was extracted from liver, spleen, kidney and lung using the Wizard^®^ Genomic Purification Kit (Promega Corporation, USA). All real-time PCRs were performed on 500 ng of genomic DNA using iQ SYBR green fluorophore (BioRad, Australia) with primers used to detect M3 (F 5’-GACCTAGCTGGCCTGGATTC-3’, R 5’-TGTACTGTTCCTCCAAC-3’), GAPDH (F 5’-GGCATCACTGCAACTCAGAA-3’, R 5’-TTCAGCTCTGGGATGACCTT-3’) and 36B4 (F 5’-CAACGGCAGCATTTATAACCC-3’, R 5’-CCCATTGATGATGGAGTGTGG-3’). Relative M3 expression was normalized to reference genes murine GAPDH or 36B4 using the ^ΔΔ^CT method.

### Circulating M3 protein levels

Circulating M3 protein was isolated from 250 μL mouse plasma using the anti-c-myc immunoprecipation kit (IP0020, Sigma-Aldrich, USA) and probed for M3 protein with a rabbit anti-M3 antibody (1:2000, kind gift from Professor Pedro Simas, Portugal), followed by a goat monoclonal anti-rabbit secondary antibody conjugated with horse-radish peroxidase (HRP) at 1:1000 (sc-2030, Santa Cruz Biotechnology, USA). Additional controls including media from AdM3 (positive) plasma from mice injected with AdGFP (negative plasma control) were run in parallel.

### Detection of GFP in liver sections

Fresh frozen livers were sectioned at 5 μm, and stained with Vector Mount for fluorescence plus DAPI (Vectorlabs, USA). Presence of GFP fluorescence in microscope images were taken using Image-Pro^®^ software.

### Aortic arch phospho p65 expression

Phosphorylated p65 levels were determined by Western immunoblotting in homogenized aortic arch tissue (30 μg). Membranes were probed with anti-phospho p65 antibody (1:1000, ab86299, Abcam, UK) followed by a goat monoclonal anti-rabbit secondary antibody conjugated with HRP at 1:1000 (sc-2030, Santa Cruz Biotechnology, USA). The membranes were then stripped and re-probed with anti-p65 (total) antibody (1:1000, ab32536, Abcam, UK) followed by the same secondary antibody as above.

### *Ex vivo* testing of chemokine activity using chemotaxis assays

Chemotaxis of human elutriated monocytes in response to mouse plasma was tested *ex vivo* using the Boyden chamber method. Monocytes were fluorescently labeled with calcein AM (5 nM, Invitrogen, USA) then re-suspended at a cell density of 5x10^5^ cells/mL in chemotaxis media (25 mM HEPES, 0.1% bovine serum albumin in RPMI media). To the bottom chambers, 600 μL of chemotaxis media and 2 μL of mouse plasma were added. To the upper chamber, 5x10^4^ cells/100 μL of monocyte suspension was placed in each well and allowed to migrate towards the lower chamber for 1 h. The number of migrated monocytes was determined as described earlier.

### Histology and immunohistochemistry

Serial transverse sections (5 μm) were taken through the aortic sinus. To quantify lesion area, 3 sections were chosen that were proximal, middle and distal to the top of the aortic sinus and stained with Milligan’s trichrome to highlight the plaque for determination of total plaque area. Collagen content was measured (green of trichrome stain) and extracellular lipid or cholesterol clefts by acellular regions of the plaque that remain following the dissolution of cholesterol crystals from degraded macrophage foam cells during the paraffin embedding process in aortic sinus lesions [11]. For macrophage immunostaining, sections were incubated overnight at 4°C with purified rat anti-mouse monoclonal Mac 3 antibody at a 1:100 dilution (550292, BD Biosciences, USA) and then incubated for 30 minutes with biotinylated anti-rat IgG affinity-purified antibody at 1:200 (BA-4000, Vector Laboratories, USA). Immune-mediated inflammatory cells within the plaque were assessed by purified rat anti-mouse CD8a (550281, BD Biosciences, USA) and biotin mouse anti-mouse CD22.2 (553382, BD Biosciences, USA) antibodies at a 1:20 dilution and then incubated similarly with biotinylated anti-rat IgG affinity-purified antibody at 1:200 (BA-4000, Vector Laboratories, USA) to determine T and B cell content respectively. Sections were stained for SMC α-actin^+^ cells using a mouse monoclonal anti-α-actin antibody conjugated to alkaline phosphatase at 1:100 (A5691, Sigma-Aldrich, USA) and visualized with Vector Red alkaline phosphatase substrate (Vector Laboratories, USA). Sections (*n* = 3/mouse) were quantified from digitized microscopic images using Image-Pro^®^ software.

### Circulating neutrophil and monocyte numbers

To investigate the effect of systemically delivered AdM3 on circulating inflammatory cells, blood from the tail vein was collected following adenoviral delivery every week up to the fourth week in the ‘rapid promotion’ model and every month up to the twelfth week in the ‘slow progression’ model. Red blood cell lysis was performed on 200 μL of blood using cell lysis buffer (BD Biosciences, USA) before being resuspended in PBS buffer. Cells were incubated with a Zombie Aqua viability stain at a 1:100 dilution (BV510 excitation) (Biolegend, USA) to label any unviable/dead cells for 30 minutes. Cells were washed and Fc receptors blocked with an Fc block (BD Biosciences, USA) for 20 minutes. Cells were washed again and then stained with a cocktail of antibodies against CD45 (1:100 rat anti-mouse CD45-APC-Cy7, BD Biosciences), Ly6-C/G (1:100 rat anti-mouse Ly6-C/G-PerCP-Cy5.5, BD Biosciences) and CD115 (1:100 rat anti-mouse CD115-APC eBioscience) for 20 minutes. Cells were washed twice and resuspended in 300 μL of HBSS buffer. Monocytes were identified as CD45^hi^CD115^hi^ and neutrophils as CD45^hi^CD115^lo^Ly6-C/G^hi^. Monocytes were further subdivided into Ly6-C/G^hi^ to distinguish between inflammatory and patrolling monocytes. The gating strategy is presented in S2 Fig. All wash steps were performed with HBSS buffer (HBSS (Sigma-Aldrich) + 0.1% BSA w/v (Sigma-Aldrich) + 5mM EDTA (Sigma-Aldrich)). All flow cytometry experiments were performed on a FACSverse (BD Biosciences, USA).

### RNA expression

Total RNA was isolated from aortic arch samples using TRI reagent. 250 ng total RNA was reverse transcribed using the iScript cDNA synthesis kit before amplification using iQ SYBR in a Cfx384 thermocycler (BioRad, Australia). The following mouse primers were used: SMC α-actin (F 5’-TGTACCAGGCATTGCTGAC-3’, R 5’-GAGGCGCTGATCCACAAAAC-3’), CD68 (F 5’-GGGGCTCTTGGGAACTACAC-3’, R 5’-GTACCGTCACAACCTCCCTG-3’), p65 (F 5’-AGTATCCATAGCTTCCAGAACC-3’, R 5’-ACTGCATTCAAGTCATAGTCC-3’). Relative gene expression was normalized to GAPDH (reference gene) using the ^ΔΔ^C_t_ method.

### ELISA immunoassays

Mouse CCL2 and CCL5 protein levels were determined in mouse plasma (75 μL for each) and mouse CCL2, CCL5 and CX_3_CL1 protein levels were determined in homogenized liver tissue (500 μg for CCL2, 10 μg for CCL5 and 1000 μg for CX_3_CL1) in lysis buffer (80 mM Tris HCl, 10 mM NaCl, 50 mM NaF, 5 mM Na_4_P_2_O_7_, 15 mM Triton-X 100) by ELISA (R&D Systems, USA). Mouse plasma diluted 1:200 was also used to determine pro-inflammatory cytokine mouse Serum Amyloid A (SAA) protein levels (10 μL for rapid promotion model and 75 μL for slow progression model) as well as undiluted mouse plasma was used to measure TNF-alpha (Tumor necrosis factor alpha) protein concentration (200 μL in both models) by ELISA (R&D Systems, USA).

### Oil Red O staining

Oil Red O (Sigma-Aldrich, USA) was utilized to identify atherosclerotic lesions in the mouse thoracic aorta. Descending thoracic aortas were stained with Oil Red O solution (0.25% Oil Red O w/v, 62.5% isopropanol v/v and 37.5% ddH_2_0 v/v) and stored in 4% paraformaldehyde until they were pinned out and imaged. Using a stereological microscope, aortas were cut open longitudinally and pinned *en face* on paraffin wax with 0.1mm insect pins (Fine Science Tools, Canada) and imaged. Lesion area was determined as a percentage of total aortic area from digitized microscopic images using Image-Pro^®^ software.

### Statistical analysis

All data are expressed as mean±SEM. All data were compared using t-test of unpaired samples assuming equal variances or a One-way ANOVA with Bonferonni’s *post hoc* test. Significance was set at *p*<0.05.

## Results

### M3 inhibits chemokine activity *in vitro*

**Fig 1:**
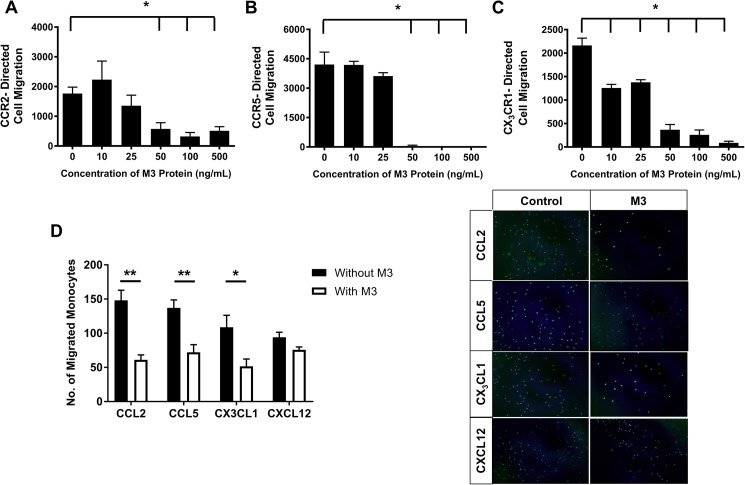
M3 inhibits chemokine activity *in vitro*. The Boyden chamber migration assay was used to assess **A.** CCR2-, **B.** CCR5- and **C.** CX_3_CR1-directed cell migration *in vitro*. 293T cells were transfected with plasmids encoding CCR2, CCR5 or CX_3_CR1. **D.** Migration of human primary monocytes towards CCL2, CCL5, CX_3_CL1 and CXCL12 in Boyden chamber migration assay. Representative images of Calcein AM labelled monocytes migrated to the underside of transwell membranes with and without purified M3 protein (100 ng/ mL) are shown. Data are mean±SEM. *p<0.05, **p<0.01.

To investigate the effects of purified M3 protein on chemokine activity *in vitro*, CCR2-, CCR5- and CX_3_CR1-directed cell migration towards their respective chemokines CCL2, CCL5 and CX_3_CL1 in the presence of M3 protein (10–500 ng/mL) was assessed. Cell migration towards purified CCL2 (Fig 1A) and CCL5 (Fig 1B) was significantly reduced with purified M3 protein at concentrations of 50, 100 and 500 ng/ mL (*p*<0.05 for all). Furthermore, M3 caused a marked decrease in CX_3_CR1-directed cell migration across all concentrations (*p*<0.05 for all, Fig 1C). Next, we tested the effect of M3 protein on the migration of human elutriated monocytes towards recombinant chemokines CCL2, CCL5, CX_3_CL1 and CXCL12. Monocyte migration towards purified CCL2, CCL5 and CX_3_CL1 was significantly reduced in the presence of M3 protein (*p*<0.05 for all, Fig 1D). M3 did not inhibit, however, the migration of monocytes towards CXCL12 (Fig 1D).

### Confirmation of gene transfer and inhibition of plasma chemokine activity by M3

**Fig 2:**
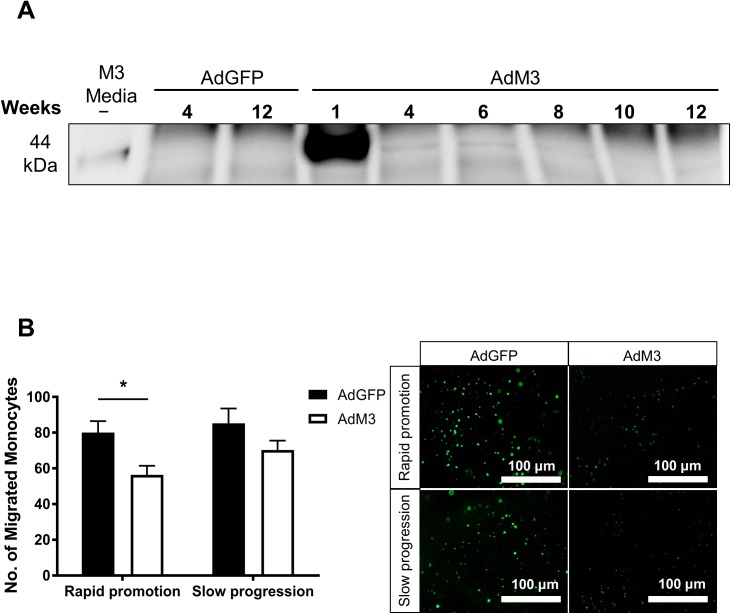
Confirmation of gene transfer and inhibition of plasma chemokine activity by M3. Two models of ‘rapid promotion’ or ‘slow progression’ of atherosclerosis were established in which a HFD or regular chow were fed and AdM3 or AdGFP were infused (*n* = 10–12/group). See “Materials and Methods” for details. Successful gene transfer and expression following adenoviral delivery was determined by Western immunoblotting. **A.** Culture media from Ad293 cells infected with AdM3 as well as plasma from mice infused with AdGFP and AdM3 were assessed from 4 to 12 weeks post adenoviral delivery following immunoprecipitation with anti-c-myc agarose beads. **B.** Chemokine activity was measured using the Boyden chamber migration assay. Calcein-AM labelled monocytes were allowed to migrate towards plasma from mice infused with AdGFP or AdM3 (*n* = 10–12/group). Data are mean±SEM. *p<0.05, ****p<0.0001.

Circulating M3 protein was detected in AdM3 mouse plasma (Fig 2A) at 1, 4, 6 and 8 weeks that was not present in the plasma of mice infused with AdGFP. The M3 plasma levels were high 1 week post-AdM3 infusion. This then dropped but was still detectable 4 weeks post-AdM3 and then appeared to remain at a similar level for ~4 more weeks. We also detected the presence of M3 protein in the media of Ad293 cells infected with (positive blot control). RTPCR measurements of viral M3 expression revealed that the gene transfer occurred predominately in the liver and not in other organs such as the spleen, kidney or lung (S1 Fig). Gene transfer was also confirmed for the AdGFP control as green fluorescent cells were detected in liver sections of AdGFP infused mice. To determine the effect of M3 expression on plasma chemokine activity *ex vivo*, a migration assay was performed in which human elutriated monocytes were allowed to migrate towards plasma from either AdM3- or AdGFP-infused mice. Monocyte migration was significantly reduced towards AdM3 mouse plasma in the ‘rapid promotion’ model (29.6%, *p*<0.05, Fig 2B). A non-significant decline in monocyte migration towards AdM3 mouse plasma was detected in the ‘slow progression’ model.

### M3 reduces atherosclerotic plaque size when the rate of plaque development is less rapid

**Fig 3:**
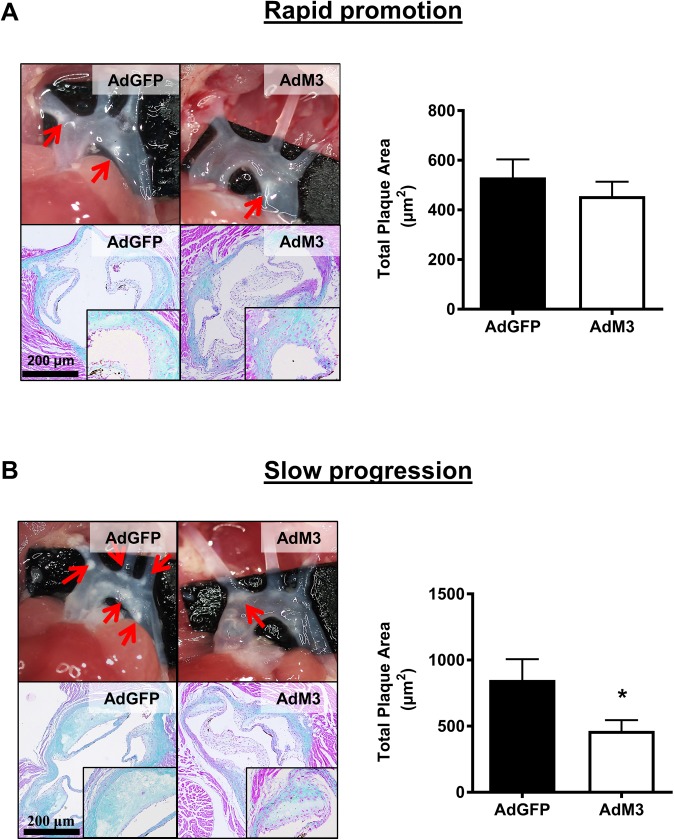
M3 reduces atherosclerotic plaque size when the rate of plaque development is less rapid. Two models of ‘rapid promotion’ or ‘slow progression’ of atherosclerosis were established in which a HFD or regular chow were fed and AdM3 or AdGFP were infused (*n* = 10–12/group). See “Materials and Methods” for details. Images are representative pictures of aortic arches (upper panels) and Trichrome stained aortic sinus sections (lower panels). Quantification of total plaque area (μm^2^) was determined from 3 sections/mouse, spanning the entire aortic sinus for **A.** the ‘rapid promotion’ model and **B.** the ‘slow progression’ model. Data are mean±SEM. *p<0.05.

There were no effects on body weight or plasma lipid levels between groups (S1 Table). In the ‘rapid promotion’ model there were no changes in atherosclerotic plaque area between mice infused with AdM3 and AdGFP (Fig 3A). However, in the slow progression model, AdM3 infused mice had significantly smaller atherosclerotic lesions (45.3%, *p*<0.05), compared to AdGFP control mice (Fig 3B).

### M3 reduces plaque macrophage content and p65 activation when the rate of plaque development is more rapid

**Fig 4:**
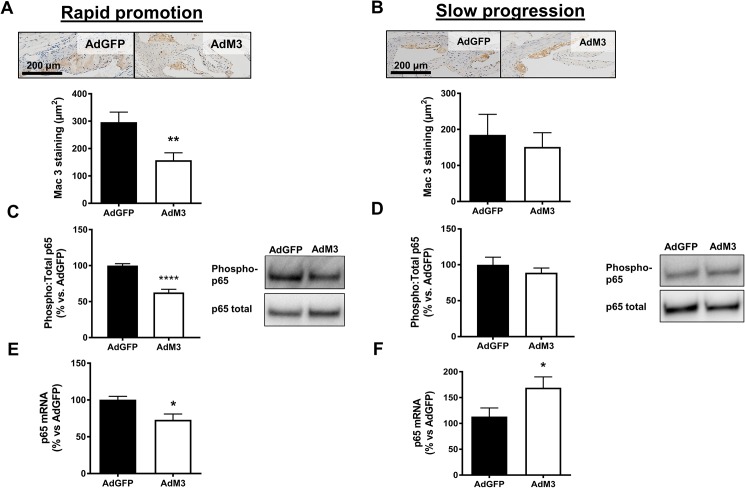
M3 reduces plaque macrophage content and p65 activation when the rate of plaque development is more rapid. Two models of ‘rapid promotion’ or ‘slow progression’ of atherosclerosis were established in which a HFD or regular chow were fed and AdM3 or AdGFP were infused (*n* = 10–12/group). See “Materials and Methods” for details. Upper panels are representative images of Mac-3^+^ macrophages (brown staining) in aortic sinus sections. Quantification of macrophage staining within plaques (μm^2^) for **A.** the ‘rapid promotion’ model and **B.** the ‘slow progression’ model. Phosphorylated p65 levels were measured in aortic arch samples for **C.** the ‘rapid promotion’ model and **D.** the ‘slow progression’ model. p65 mRNA levels were determined in aortic arch samples for **E.** the ‘rapid promotion’ model and **F.** the ‘slow progression’ model. Data expressed as mean±SEM. *p<0.05, **p<0.01, ****p<0.0001.

Histological analysis showed that macrophage content in the aortic sinus of AdM3 mice was strikingly reduced (46.8%, *p*<0.01) in the ‘rapid promotion’ model (Fig 4A). In contrast, there was no change in plaque macrophage content in the ‘slow progression’ model (Fig 4B).

Consistent with the reduction in macrophage content, there was a significant reduction in levels of phosphorylated (phospho) p65, the active subunit of NF-κB, in the aortic arches of AdM3 mice (37.3%, *p*<0.0001) in the ‘rapid promotion’ model (Fig 4C). There were, however, no changes in phospho p65 in the ‘slow progression’ model (Fig 4D). These findings were further supported by a decrease in the aortic arch mRNA levels of p65 of AdM3 mice (27.2%, *p*<0.05) in the ‘rapid promotion’ model (Fig 4E). Conversely, in the ‘slow progression’ model there was an increase in p65 (33%, *p*<0.05) in the aortas of AdM3-infused mice (Fig 4F). There were no changes in plaque T or B cell content between groups. Neither were there changes in plaque collagen or extracellular lipid content (S3 Fig).

### M3 prevents a decrease in inflammatory monocytes in the rapid promotion model four weeks’ post-gene transfer

**Fig 5:**
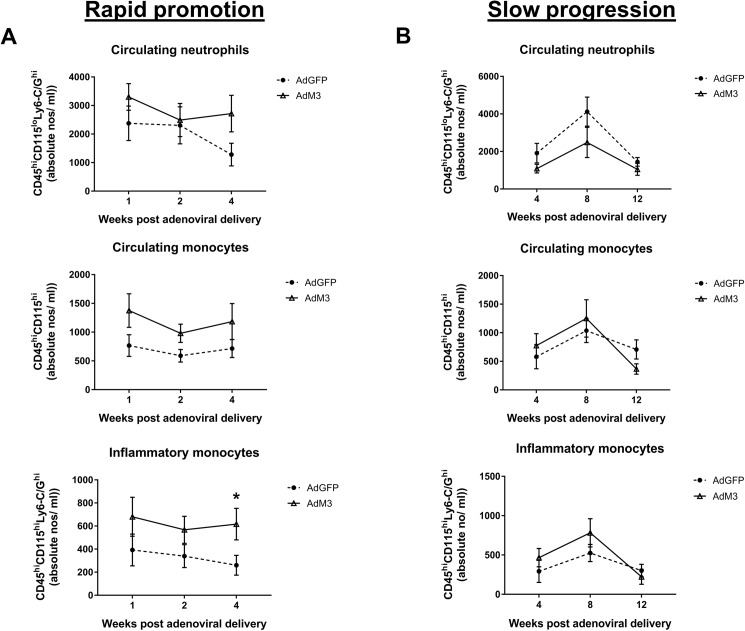
M3 prevents a decrease in inflammatory monocytes in the rapid promotion model four weeks’ post-gene transfer. Two models of ‘rapid promotion’ or ‘slow progression’ of atherosclerosis were established in which a HFD or regular chow were fed and AdM3 or AdGFP were infused (*n* = 7/group). See “Materials and Methods” for details. Circulating neutrophils (CD45^hi^CD115^lo^Ly6-C/G^hi^) and monocytes (CD45^hi^CD115^hi^) were measured for Ly6-C/G by flow cytometry every week up to 4 weeks in **A.** the ‘rapid promotion’ model and every month up to 12 weeks in **B.** the ‘slow progression’ model.

To assess the effect of M3 on circulating leukocytes, neutrophils and monocytes were measured longitudinally in the whole blood of both models (Fig 5A and 5B). At all times points after adenoviral delivery there were no significant differences in the total number of circulating neutrophils or monocytes in either model. However, 4 weeks’ post-injection AdM3 treated mice had a 57.8% (*p*<0.05) increase in inflammatory monocytes in the rapid promotion model, compared to AdGFP control mice. This suggests that AdM3 is preventing a decrease in inflammatory monocytes.

### M3 increases plaque SMC content in the ‘slow progression’ atherosclerosis model

**Fig 6:**
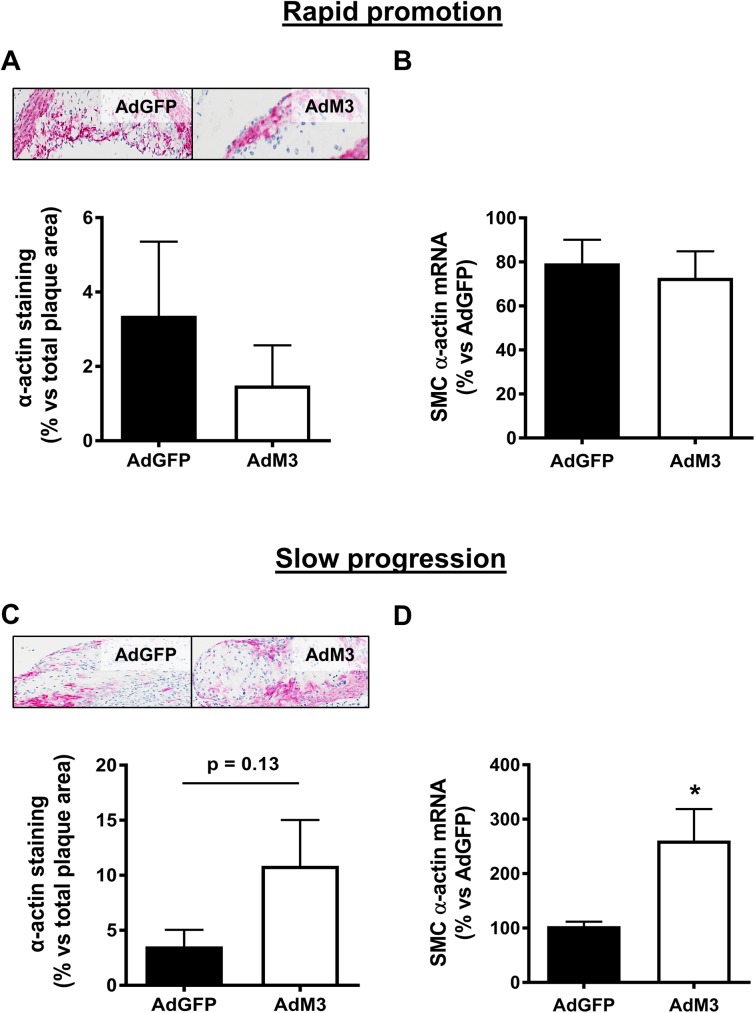
M3 increases plaque SMC content in the ‘slow progression’ atherosclerosis model. Two models of ‘rapid promotion’ or ‘slow progression’ of atherosclerosis were established in which a HFD or regular chow were fed and AdM3 or AdGFP were infused (*n* = 10–12/group). See “Materials and Methods” for details. Images are representative sections of plaque α-actin^+^ SMCs (red staining) in aortic sinuses. SMC α-actin staining was quantified as a percentage of total aortic sinus plaque area and SMC α-actin mRNA levels were determined in aortic arch samples for the ‘rapid promotion’ model **(A—B)** and the ‘slow progression’ model **(C—D)**. Data expressed as mean±SEM. *p<0.05.

No significant differences were seen in plaque SMC content in the ‘rapid promotion’ model (Fig 6A). Consistent with this, there were no changes in SMC α-actin mRNA expression in the aortas of AdM3 infused mice (Fig 6B). However, in the ‘slow progression’ model there was a trend for an increase in plaque SMC content with M3 overexpression (67.4%, *p* = 0.13, Fig 6B), which was supported by a significant increase in aortic SMC α-actin mRNA expression (60.3%, *p*<0.05) in this model (Fig 6D).

### M3 reduces lipid deposition in descending aortas

**Fig 7:**
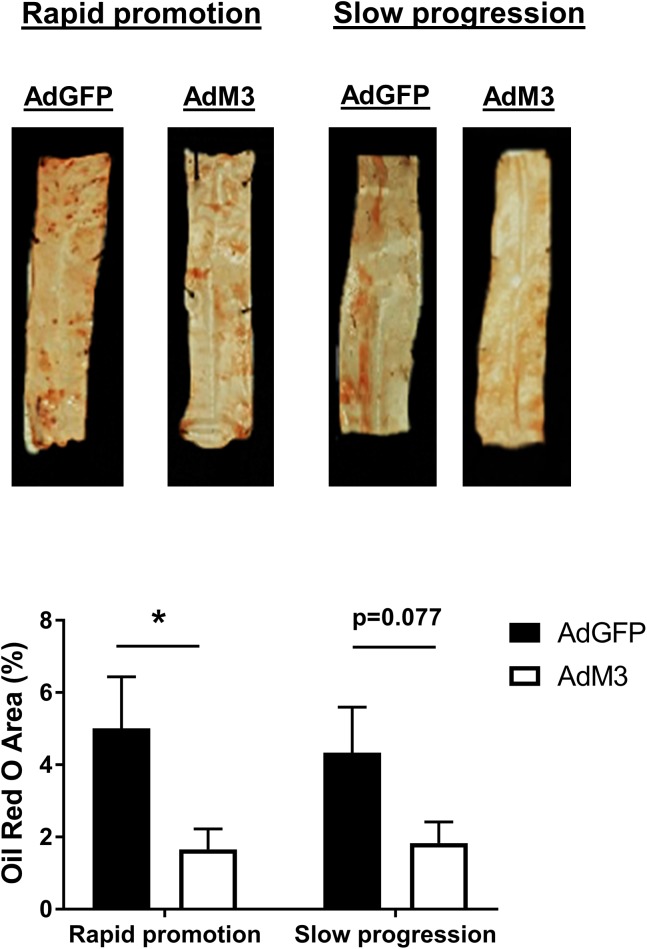
M3 reduces lipid deposition in descending aortas. Two models of ‘rapid promotion’ or ‘slow progression’ of atherosclerosis were established in which a HFD or regular chow were fed and AdM3 or AdGFP were infused (*n* = 10–12/group). See “Materials and Methods” for details. Images are representative sections of Oil Red O stained descending thoracic aortas. Oil Red O staining was quantified as a percentage of total thoracic aorta area for the ‘rapid promotion’ model and the ‘slow progression’ model. Data are mean±SEM. *p<0.05.

To determine the effects of M3 on lipid deposition, excised aortas were stained with Oil Red O. Lipid content in thoracic aortas of AdM3-treated mice was reduced in both models, reaching significance in the rapid promotion model (66.9%, *p*<0.05) (Fig 7) while a trend was seen in the slow progression model (*p* = 0.077).

### M3 modulation of chemokine levels in the plasma and tissue

**Fig 8:**
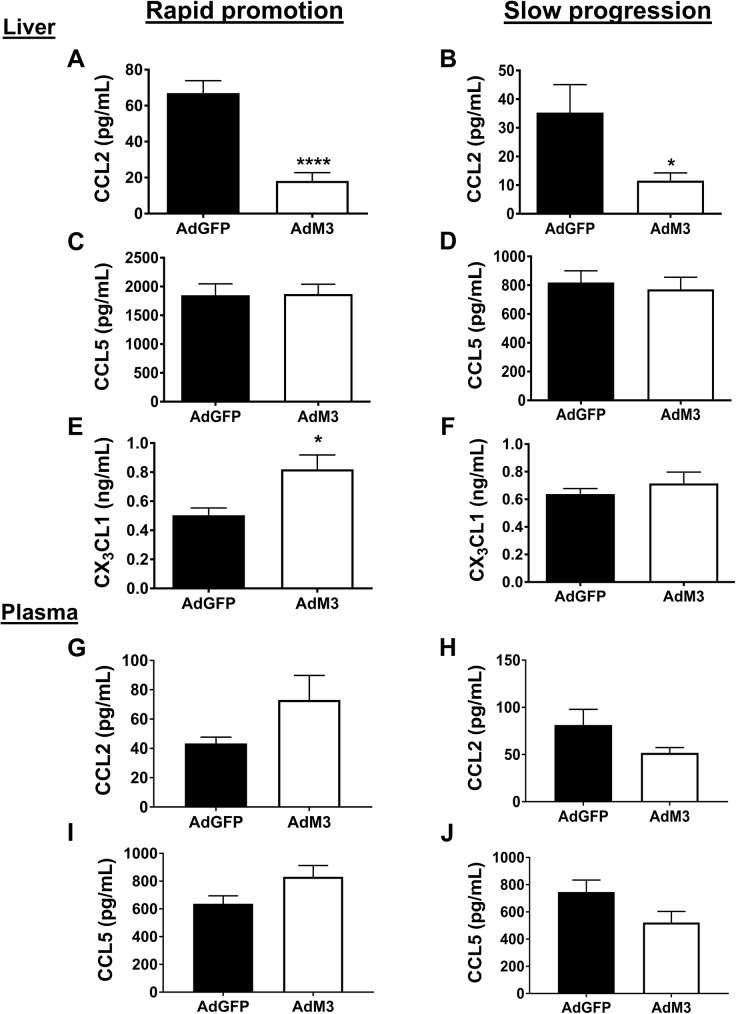
M3 regulation of chemokine levels in the plasma and tissue. Two models of ‘rapid promotion’ or ‘slow progression’ of atherosclerosis were established in which a HFD or regular chow were fed and AdM3 or AdGFP were infused (*n* = 10–12/group). See “Materials and Methods” for details. CCL2, CCL5 and CX_3_CL1 protein levels were assessed in the liver **(A-F)** and CCL2 and CCL5 in the plasma **(G-J)** by ELISAs. Data are mean±SEM. *p<0.05, ****p<0.0001.

Systemic overexpression of M3 was found to strikingly reduce CCL2 liver protein levels in both models (rapid promotion: 72.8% and slow progression: 67.3%, *p*<0.05 for all, Fig 8A and 8B). No significant differences were seen in CCL5 liver protein levels with M3 in either model (Fig 8C and 8D). However, M3 increased CX_3_CL1 liver protein levels (37.8%, *p*<0.05) in the rapid promotion model but not in the slow progression model (Fig 8C). Assessment of circulating chemokines found that there were no significant changes in either CCL2 or CCL5 in the plasma for either model of atherosclerosis progression with M3 (Fig 8G–8J). There were no changes in the plasma levels of SAA or TNF-α between groups (S4 Fig).

## Discussion

In this study, we investigated the effectiveness of the broad-spectrum chemokine inhibitor M3 on atherosclerosis progression in two models with differing rates of plaque development including: a (1) HFD-fed ‘rapid promotion’ model and (2) chow-fed ‘slow progression’ model. M3 exhibited varying effects in the two different models. Adenoviral overexpression of M3 significantly reduced lipid deposition in the descending aorta in the ‘rapid promotion model’ and reduced macrophage content in aortic sinus plaques. M3 protein also reduced aortic sinus lesion size and increased plaque SMC content in the ‘slow progression’ model. The changes in plaque size and macrophage content by M3 in the two models are likely to have been mediated through the inhibition of chemokine activity. *In vitro* studies found that M3 protein suppressed chemokine-receptor directed migration and human primary monocyte migration towards CCL2, CCL5 and CX_3_CL1, and plasma samples from AdM3 mice induced less monocyte migration than plasma from AdGFP viral control mice. Our findings suggest that variations in the aggressiveness of disease onset influence the effectiveness of M3 to alter plaque composition and limit atherosclerosis. This indicates that competitive inhibition of a few key chemokines across all the chemokine subclasses with M3 may not be as robust as inhibiting an entire class of chemokines (e.g. the CC-chemokine class with 35K) or the complete removal of a single chemokine/chemokine receptor (e.g. murine knockout models) for the suppression of atherosclerosis.

In the present study, we have demonstrated that chemokine-receptor directed migration towards purified CCL2, CCL5 and CX_3_CL1 was significantly reduced in response to increasing concentrations of purified M3 protein. In support of this finding, we also showed that cell migration of human monocytes towards key inflammatory chemokines was suppressed in the presence purified M3 protein, but this effect was not observed in migration towards CXCL12, a chemoattractant that is not inhibited by M3. This is consistent with studies that have reported M3 inhibition of CCL2, CCL5 and CX_3_CL1 but not CXCL12 [16]. Furthermore, we found that M3 protein, secreted into the plasma of AdM3-injected mice, suppressed plasma chemokine activity, as fewer monocytes migrated towards plasma from AdM3-injected mice. This reduction in plasma chemokine activity was significant in mice of the ‘rapid promotion’ model and is comparable with other studies using a similar chemokine blockade approach with the CC-chemokine class inhibitor ‘35K’ [11]. The more modest reduction in plasma chemokine activity in the ‘slow progression’ model is likely due to the longer gene transfer period post-AdM3 infusion. Plasma from the mice in the ‘slow progression’ model was collected 12-weeks post-AdM3 delivery, compared to 4-weeks for the ‘rapid promotion’ model. After 12-weeks the expression of M3 may be declining. This is indicated by our detection of M3 protein using Western blotting in which there is a decline after one week and the reduction in AdM3 gene expression in the livers of the ‘slow progression’ mice. Despite this, the levels of M3 over the 12-week time period were sufficient to significantly reduce atherosclerotic lesion size in the aortic sinus and increase plaque SMC content.

This study found that M3 was more effective at inhibiting a slower rate of atherosclerosis than a higher rate of atherosclerosis. The chow-fed ‘slow progression’ model is more representative of physiological plaque development than HFD-fed rapid plaque progression, suggesting that M3 may be an effective strategy to limit atherosclerosis. In support of this there was also a reduction in lipid deposition in the descending thoracic aorta of the mice of the ‘rapid promotion’ model, which is also a site in which the progression of atherosclerosis is slower. The lack of effect on plaque size in the ‘rapid promotion’ model at the site of the aortic sinus, in which plaque formation is relatively fast, indicates that M3 may not be as effective as other chemokine inhibition strategies that have managed to suppress the more aggressive atherosclerosis generated by HFD feeding [8–11].

It was counter-intuitive to find no changes in lesions size in the aortic sinus in the HFD-fed ‘rapid promotion’ model, despite a reduction in plaque macrophage content. It was postulated that this may be due to an increase in the infiltration of other cell types such as T cells and B cells [20]. This is possible, as M3 does not inhibit the chemokines that direct T and B cell migration (e.g. CCL12, CCL19, CCL25) [21]. We did not find, however, any changes in plaque T or B cell content with M3. The effectiveness of M3 to decrease plaque macrophage content in the ‘rapid promotion’ model but not in the ‘slow progression’ model may be attributed to the higher rate of macrophage recruitment in HFD-fed mice, therefore enabling a greater opportunity for inhibition with M3. Support for a role of M3 in the inhibition on macrophage recruitment has also been identified in the gut [22]. The potential contribution from circulating and inflammatory monocytes was also assessed in an attempt to explain these observations. We found that 4 weeks post adenoviral delivery, M3 unexpectedly prevented a decrease in the proportion of circulating inflammatory monocytes in the ‘rapid promotion’ model, compared to the AdGFP control. This suggests that a higher number of monocytes would infiltrate the neointima leading to an increase in macrophage content. The opposite, however, occurred and there was a reduction in plaque macrophage content. We hypothesize therefore that the inhibition of key chemokines by M3 (e.g. CCL2, CCL5) prevents the adherence of inflammatory monocytes to the endothelium, allowing them to accumulate in the circulation, rather than traversing the endothelium and entering the plaque.

Although our observations are spread over two different models with varying rates of plaque development, the reduction in plaque size in the aortic sinus and the descending aorta, and macrophage content via chemokine inhibition with M3 are consistent with previous animal knockout and transgenic studies, as well as double decoy chemokine studies. The knockout and transgenic studies provide convincing proof that individual chemokine-chemokine receptor signalling pathways are important in atherosclerosis, particularly the CCL2/CCR2 [8, 17], CCL5/CCR5 [23] and CX_3_CL1/CX_3_CR1 [24] pathways. However, in these studies only a single chemokine or chemokine receptor was targeted, a strategy which is likely to be limited by redundancy in chemokine signalling. In a different approach, adenoviral-mediated gene transfer of the broad-spectrum CC-chemokine inhibitor 35K was found to cause an 85% reduction in macrophage recruitment and a 55% decrement in aortic sinus lesion size, compared with its controls in HFD-fed apoE^-/-^ mice [11]. This suggests that overcoming redundancy issues, at least in the case of the CC-chemokine class, can be a very effective strategy for inhibiting atherosclerosis. Comparing the chemokine binding proteins M3 and 35K, our results suggest that targeting fewer key chemokines from all four chemokine subfamilies, as with M3, may not be as robust as blocking the entire CC-chemokine class, as with 35K, for the suppression of atherosclerosis. With its ability to block chemokines from other classes such as the CX_3_C class, M3 may, however, be advantageous in pathologies such as thrombosis and restenosis. CX_3_CL1 is reported to increase SMC cell-cell adhesion of leukocytes and promote aortic SMC proliferation–both key drivers of neointimal hyperplasia-post stent deployment [25]. Consistent with this, the development of intimal hyperplasia following wire injury is suppressed in the M3 transgenic mouse [15]. CX_3_CL1 also plays a role in the activation of platelets, which increase inflammatory cell adhesion to the endothelium and promote thrombus formation [26]. Therefore, whilst the effects of M3 on atherosclerosis are more modest than other strategies it may be highly efficacious in preventing other inflammatory-driven pathologies.

Analysis of chemokine protein levels in mouse liver tissue showed that M3 strikingly reduced CCL2 protein concentrations in both models with no effect on CCL5. There were also no significant changes in circulating CCL2 and CCL5 in the plasma. These results suggest that M3 is binding CCL2 in the tissues and then sequestering it into the circulation where it is then cleared [27]. M3 appears to have had no effect in CCL5 *in vivo*, despite our *in vitro* data showing M3 inhibits CCR5-directed cell migration and other studies demonstrating the binding of M3 to CCL5 [28]. It has been reported that M3 binds CCL2 with a significantly higher affinity than CCL5, which is likely to be a contributing factor to this apparent lack of effect [28]. There were no changes in circulating chemokine levels, despite a reduction in chemokine activity. It may have been expected that circulating chemokine levels would decrease, however, previous studies using chemokine binding proteins support our findings and have found either no change or increases in circulating chemokines levels [11, 29]. It has been suggested that this is due to compensatory increases in chemokine expression in response to the inhibition, however, the new chemokines are likely to be rapidly bound to their inhibitor and inactive, then cleared or remain bound in the circulation [11]. Furthermore, it has been shown that chemokines bound to chemokine binding proteins are inactive, yet still detectable by ELISA [27]. In contrast to the other chemokines, CX_3_CL1 protein levels increased in the liver in AdM3 infused mice. CX_3_CL1 is a unique membrane-bound chemokine and is unlikely to be sequestered into the circulation but rather remain at the site of the liver, unable to be cleared when bound to M3 [30]. Inhibition of CX_3_CL1 by M3 in the liver, the site at which the gene transfer occurs, may be causing a compensatory increase in CX_3_CL1 liver expression, however, the CX_3_CL1 which is present in higher levels may not be active due to being bound to M3 protein.

Changes were detected in the aortic arch expression of the active subunit of NF-κB (p65), a key transcription factor in the promotion of inflammation and atherosclerosis. There was a significant decrease in aortic arch phospho p65 levels and mRNA expression in the ‘rapid promotion’ model, but no changes in phospho p65 and an increase in p65 mRNA in the ‘slow progression’ model. Genetic evidence in mice has revealed complex roles for NF-κB in inflammation that suggest both pro- and anti-inflammatory roles for the NF-κB pathway [31]. While it regulates pro-inflammatory cytokine production, leukocyte recruitment, and cell survival, NF-κB can also promote leukocyte apoptosis and contribute to the resolution of inflammation [32]. It is also suggested that NF-κB contributes to the feedback control of inflammation to affect the magnitude and duration of the inflammatory response [33]. Furthermore, the levels of inflammation were likely to be higher in the HFD-fed ‘rapid promotion’ model, as shown by our measurements of SAA, providing a greater opportunity for M3 to inhibit p65. This result also parallels the reduction in plaque macrophage content in AdM3-infused mice seen exclusively in the ‘rapid promotion’ model.

Four weeks of HFD feeding induced lesions with very low levels of α-actin positive SMC as it is likely too early in the atherosclerotic process for significant smooth muscle and/or collagen remodelling to have occurred after AdM3 gene transfer, making it difficult for the differences to be detected. However, an increase of SMC content in the ‘slow progression’ model suggests that M3 may be improving plaque stability in a more long-term disease progression. The characterization of other components of the plaque that are representative of plaque stability including collagen content and extracellular lipid content were, however, unchanged by M3 protein.

**Fig 9:**
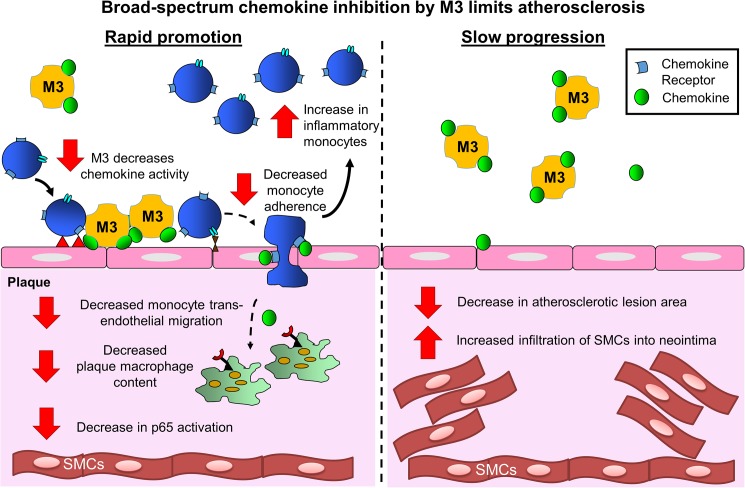
Summary schematic comparing effects of broad-spectrum inhibition by M3 on atherosclerosis. The effects of broad-spectrum chemokine inhibition by M3 was demonstrated via two models of atherosclerosis—‘rapid promotion’ and ‘slow progression’. In the rapid promotion model M3 inhibits chemokine activity, causing suppression of inflammatory monocytes, reducing adherence to the endothelium so they accumulate in the circulation rather than enter the plaque. This leads to a reduction in plaque macrophages and a suppression in lipid deposition in the descending thoracic aorta, which develops later, but not in the aortic sinus that would contain plaque of a more advanced stage. In the ‘rapid promotion’ model, aortic phosphorylated p65 was lower which may also have contributed to the reduction in plaque macrophages. In the more gradual slower progressive model, we saw an increase in plaque SMCs, a marker of improved plaque stability, with an overall reduction in atherosclerotic lesion area in the aortic sinus and descending thoracic aorta. Despite the decrease in lesion area, there was no effect on circulating monocytes or plaque macrophage content. This may be explained by the decline in M3 protein and activity at the later time points of this study and the overall lower levels of inflammation in this chow-fed model.

Adenoviral overexpression of M3 protein in apoE^-/-^ mice has varying effects depending on the rate of plaque progression (Fig 9). We propose that in the rapid promotion model M3 inhibits chemokine activity, resulting in the suppression of inflammatory monocytes adhering to the endothelium, causing them to accumulate in the circulation rather than traverse the endothelium and enter the plaque. This leads to a reduction in plaque macrophages and a suppression in lipid deposition in the descending thoracic aorta, which develops later, but not in the aortic sinus that would contain plaque of a more advanced stage. In this more aggressive inflammatory model, aortic phosphorylated p65 was lower which may also have contributed to the reduction in macrophage content. In the more gradual slower progressive model, we saw an increase in plaque SMCs indicating improved plaque stability with M3, with an overall reduction in atherosclerotic lesion area in the aortic sinus and descending thoracic aorta. Despite the decrease in lesion area, overexpression of M3 in this model had no effect on circulating monocytes or plaque macrophage content. The mechanisms for this are unclear but may be explained by the decline in M3 protein levels at the later time points of this study (represented by the attenuated inhibition of chemokine activity) and the overall lower levels of inflammation in this chow-fed model.

## Conclusion

In summary, adenoviral-mediated overexpression of M3 suppresses chemokine activity *in vitro* and *in vivo*. Chemokine inhibition by M3 caused different effects on atherosclerotic plaque size and composition that was dependent on the rate of plaque progression. These findings support a role for broad-spectrum chemokine inhibition with M3 to limit atherosclerosis, but consideration of the aggressiveness of the disease may need to be considered for maximum efficacy.

## Supporting information

S1 Fig Confirmation of successful *in vivo* gene transfer and *ex vivo* inhibition of chemokine activity by M3. Successful gene transfer and expression following adenoviral delivery was determined *in vivo*. **A.** M3 viral DNA in liver (Li), spleen (S), kidney (K) and lung (Lu) tissues was detected by real-time PCR. **B.** Livers of AdGFP and AdM3 infused mice were sectioned (5 μm) and viewed for green fluorescence. Data are mean±SEM. *p<0.05, **p<0.01,****p<0.0001, *n* = 10–12 mice/treatment group.
(TIF)

S2 Fig Gating strategy used in flow cytometry analysis to detect different subsets of lymphocytes. In this sample gating, cells were first gated for lymphocytes (FSC-A vs. SSC-A). Lymphocytes were then analysed for their uptake of the Zombie Aqua viability stain (BV510 excitation) to determine live vs. dead cells. Viable cells were further selected for singlets by gating sequentially first on a FSC-H vs. FSC-W and then on a SSC-H vs. SSC-W plot. **A.** Single cells were gated to determine CD45^hi^CD115^hi^ monocytes and **B.** further subdivided into Ly6-C/G^hi^ or Ly6-C/G^lo^ to distinguish between inflammatory and patrolling monocytes respectively. **C.** Neutrophils were determined as CD45^hi^CD115^lo^Ly6-C/G^+^ cells.
(TIF)

S3 Fig Plaque T Cell, B Cell, collagen and cholesterol cleft content. Histological analysis was performed on aortic sinus sections. **A.** CD8 positive T cells and **B.** CD22 positive B cells, **C.** collagen and **D.** cholesterol clefts. Data expressed as mean±SEM, *n* = 10–12 mice/treatment group.
(TIF)

S4 Fig Assessment of pro-inflammatory cytokines mouse SAA and TNF-alpha in plasma. Levels of pro-inflammatory cytokines were measured in mouse plasma. Data expressed as mean±SEM, *n* = 10–12 mice/treatment group.
(PPTX)

S1 Table Plasma lipids and body weight measures. Mouse plasma lipid concentrations were determined enzymatically using a commercial kit. The mean weights of each treatment group of animals at the time of sacrifice were recorded for both the **A.** rapid promotion and **B.** slow progression model. Data expressed as mean±SEM, *n* = 10–12 mice/treatment group.
(PPTX)
