# Supplementary figures and images for: Chemokine binding protein ‘M3’ limits atherosclerosis in apolipoprotein E-/- mice
Source: PLoS One. 2017 Mar 10;12(3):e0173224. doi: 10.1371/journal.pone.0173224 (PMC5345809; doi:10.1371/journal.pone.0173224)

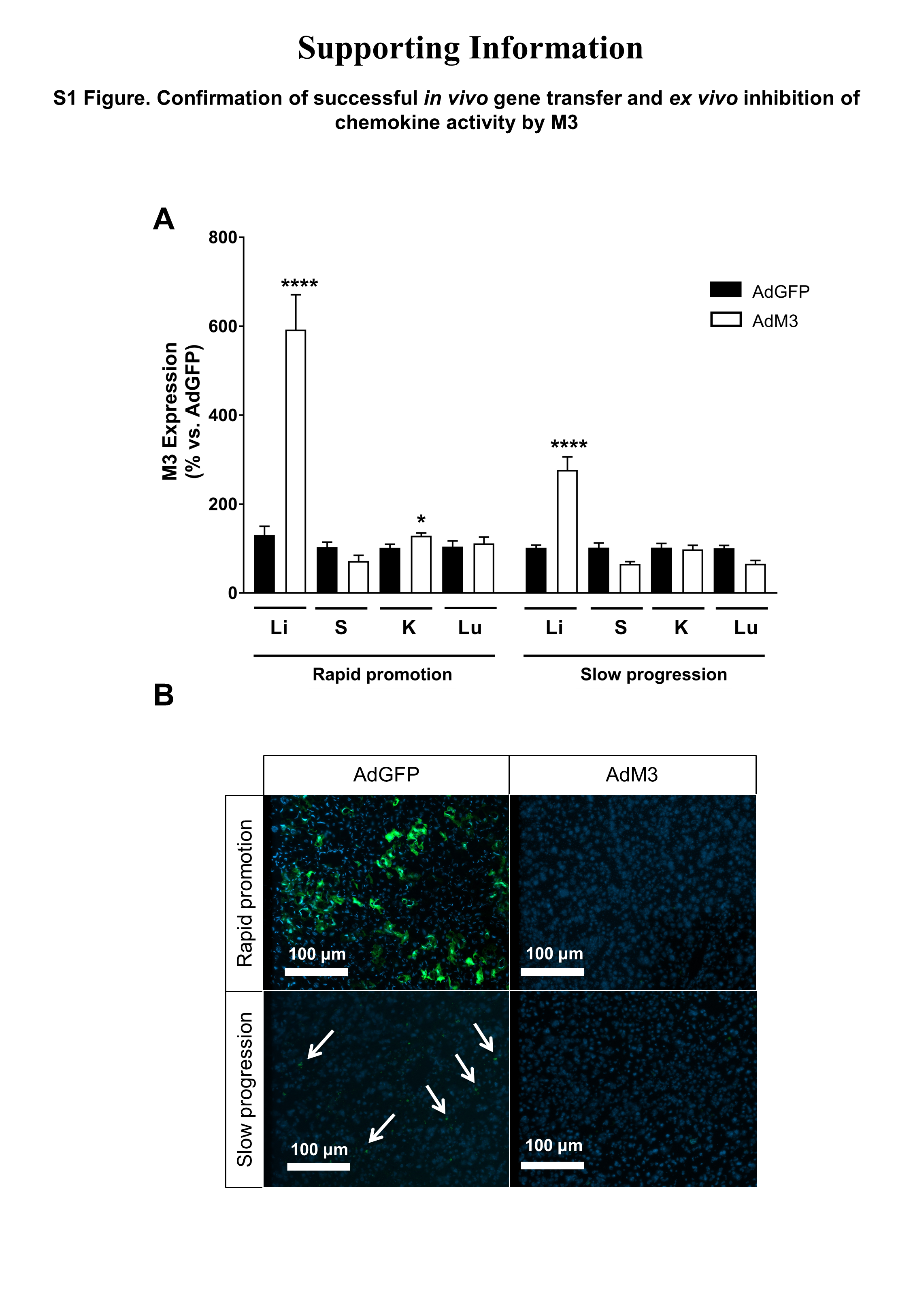

Supplement: S1 Fig — Successful gene transfer and expression following adenoviral delivery was determined in vivo. A. M3 viral DNA in liver (Li), spleen (S), kidney (K) and lung (Lu) tissues was detected by real-time PCR. B. Livers of AdGFP and AdM3 infused mice were sectioned (5 μm) and viewed for green fluorescence. Data are mean±SEM. *p<0.05, **p<0.01,****p<0.0001, n = 10–12 mice/treatment group. (TIF) [file pone.0173224.s001.TIF]

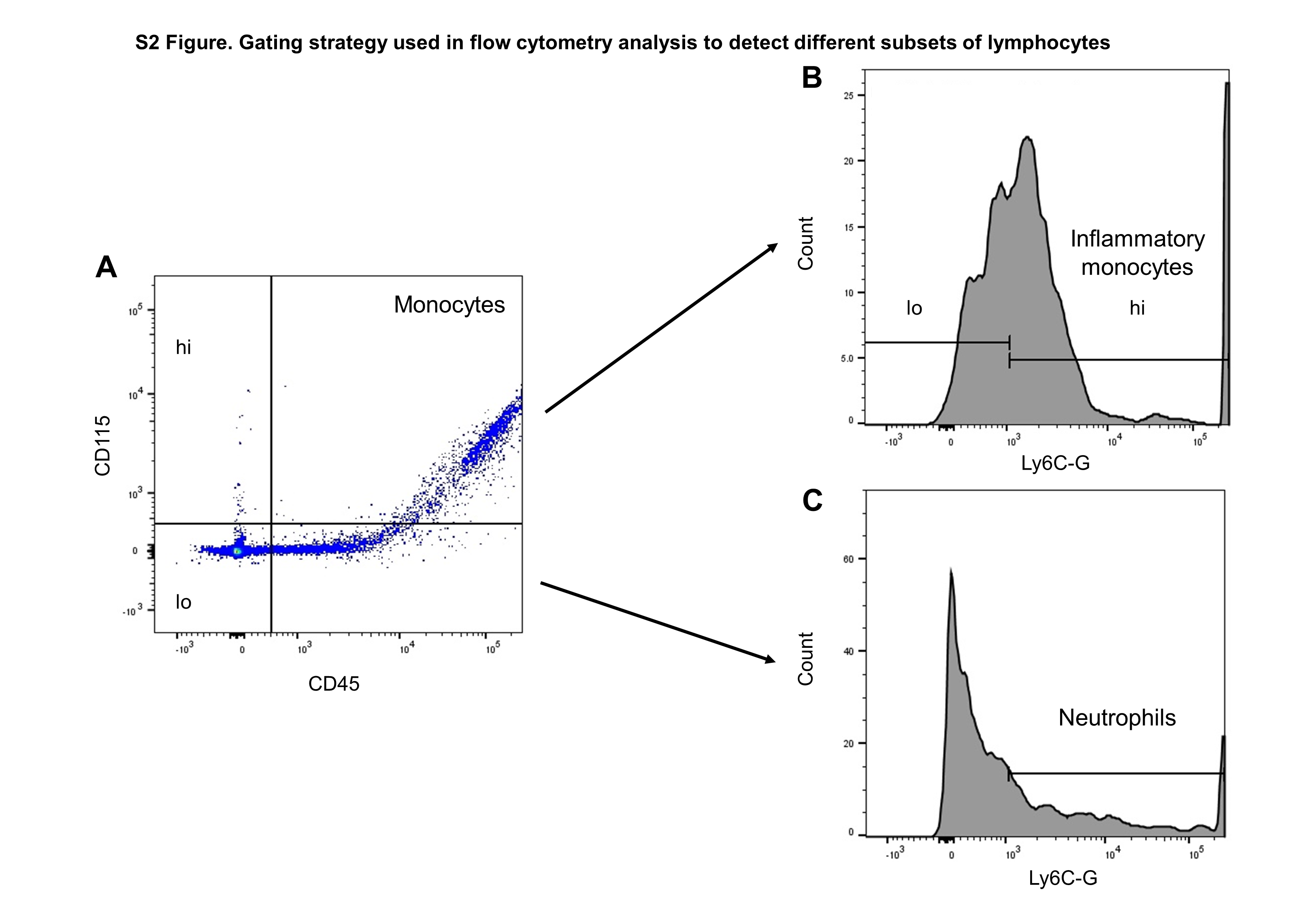

Supplement: S2 Fig — In this sample gating, cells were first gated for lymphocytes (FSC-A vs. SSC-A). Lymphocytes were then analysed for their uptake of the Zombie Aqua viability stain (BV510 excitation) to determine live vs. dead cells. Viable cells were further selected for singlets by gating sequentially first on a FSC-H vs. FSC-W and then on a SSC-H vs. SSC-W plot. A. Single cells were gated to determine CD45hiCD115hi monocytes and B. further subdivided into Ly6-C/Ghi or Ly6-C/Glo to distinguish between inflammatory and patrolling monocytes respectively. C. Neutrophils were determined as CD45hiCD115loLy6-C/G+ cells. (TIF) [file pone.0173224.s002.TIF]

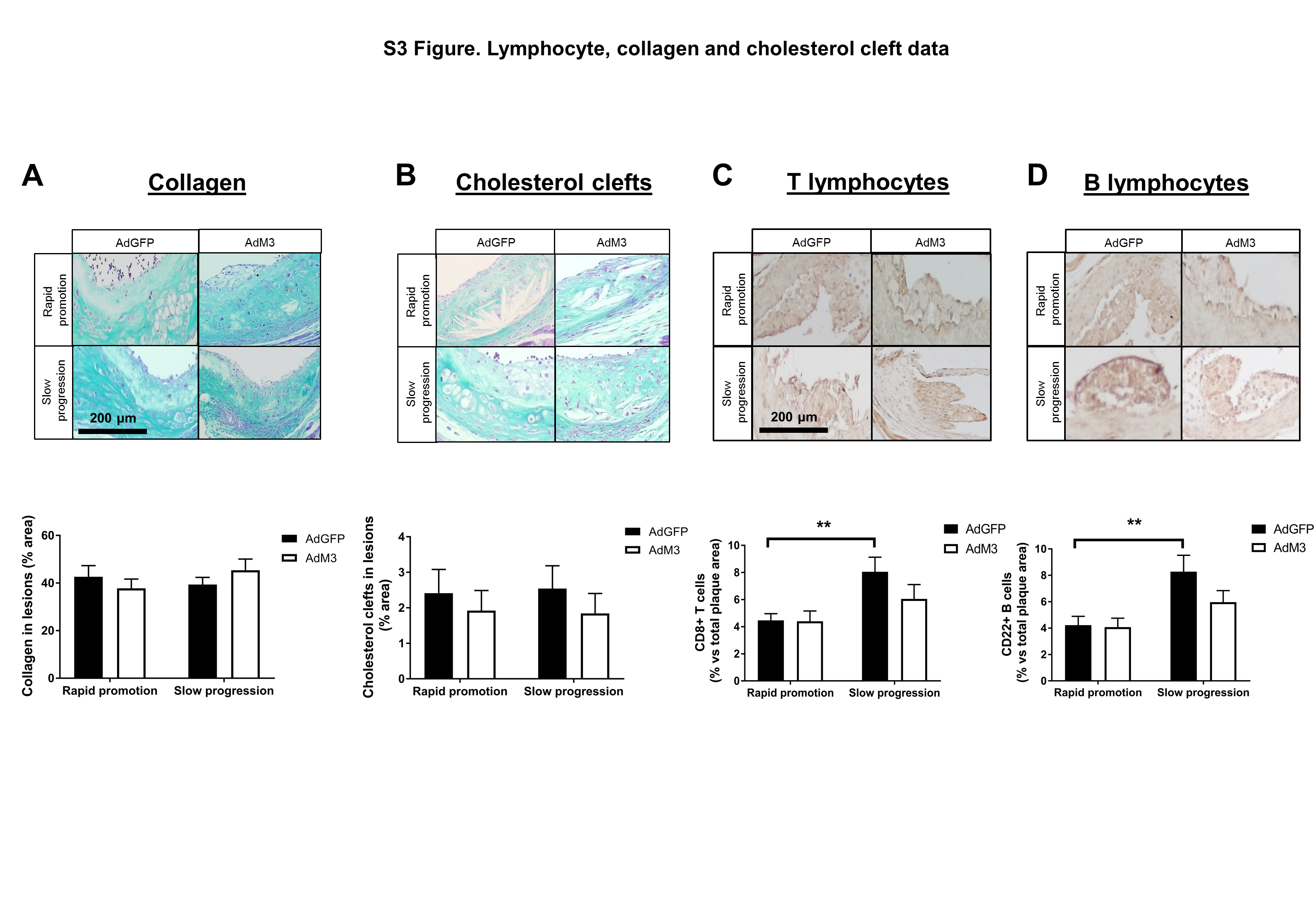

Supplement: S3 Fig — Histological analysis was performed on aortic sinus sections. A. CD8 positive T cells and B. CD22 positive B cells, C. collagen and D. cholesterol clefts. Data expressed as mean±SEM, n = 10–12 mice/treatment group. (TIF) [file pone.0173224.s003.TIF]

## Slide 1
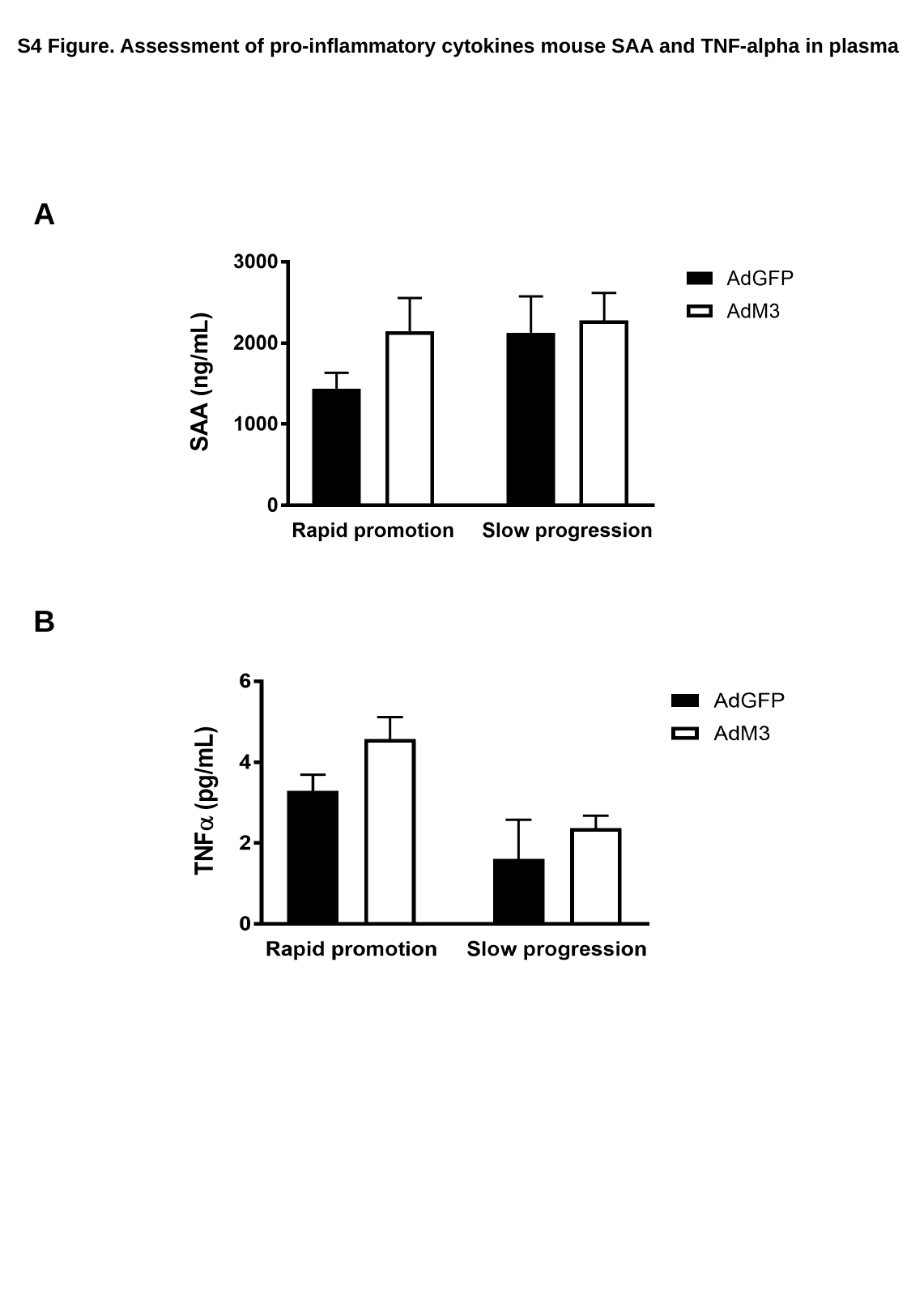

S4 Figure. Assessment of pro-inflammatory cytokines mouse SAA and TNF-alpha in plasma
A
B

Supplement: S4 Fig — Levels of pro-inflammatory cytokines were measured in mouse plasma. Data expressed as mean±SEM, n = 10–12 mice/treatment group. (PPTX) [file pone.0173224.s004.pptx]
