# Supplementary material for: Chemokine binding protein ‘M3’ limits atherosclerosis in apolipoprotein E-/- mice
Source: PLoS One. 2017 Mar 10;12(3):e0173224. doi: 10.1371/journal.pone.0173224 (PMC5345809; doi:10.1371/journal.pone.0173224)
Supplement: S1 Table — Mouse plasma lipid concentrations were determined enzymatically using a commercial kit. The mean weights of each treatment group of animals at the time of sacrifice were recorded for both the A. rapid promotion and B. slow progression model. Data expressed as mean±SEM, n = 10–12 mice/treatment group. (PPTX) [file pone.0173224.s005.pptx]

## Slide 1
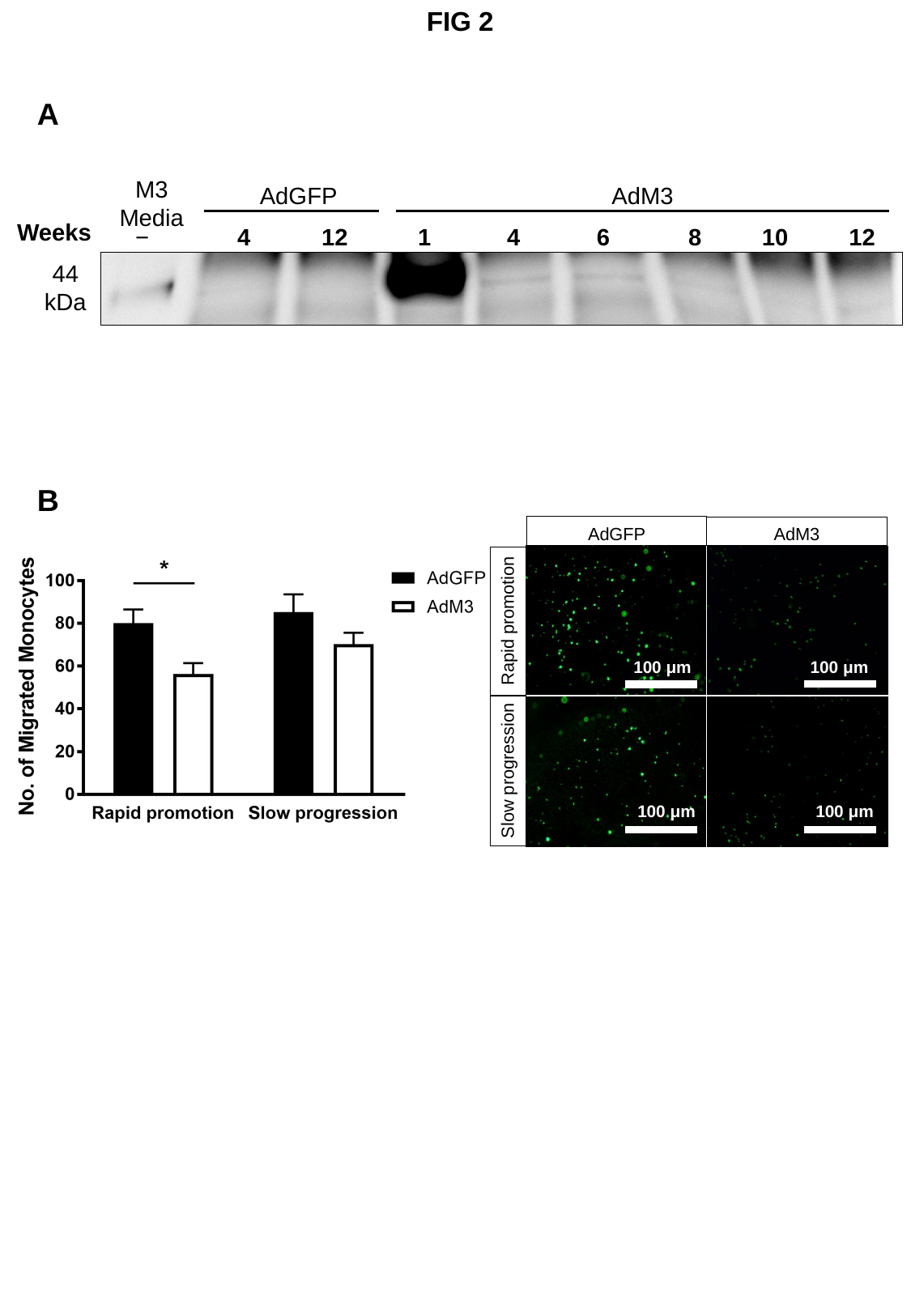

FIG 2
A
M3 Media
AdGFP
AdM3
Weeks
−
4
12
1
4
6
8
10
12
44 kDa
B
AdGFP
AdM3
Rapid promotion
C
100 μm
100 μm
Slow progression
100 μm
100 μm

## Slide 2
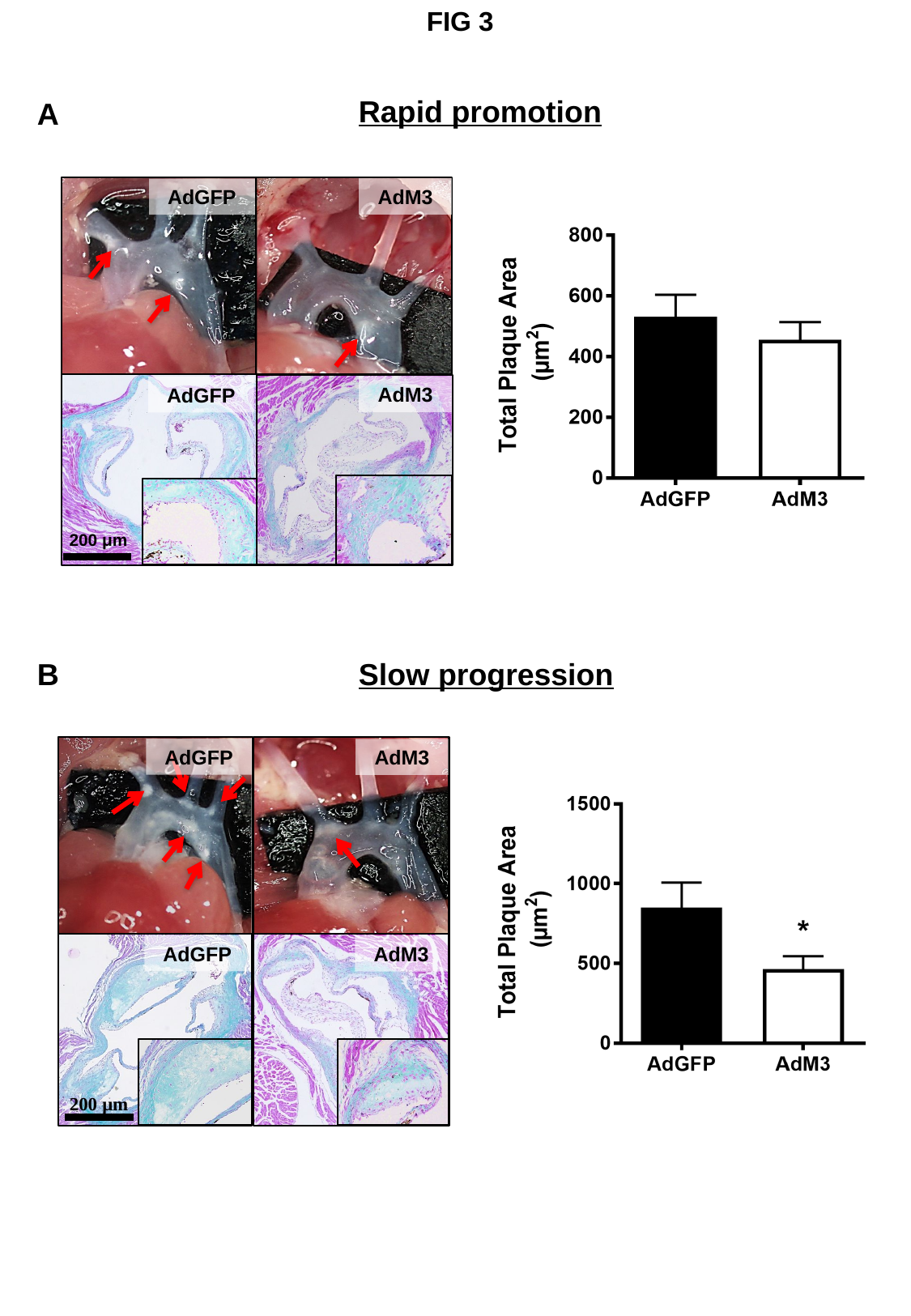

FIG 3
Rapid promotion
A
AdM3
AdGFP
AdGFP
AdM3
AdGFP
200 μm
B
Slow progression
AdGFP
AdM3
AdGFP
AdM3
200 μm

## Slide 3
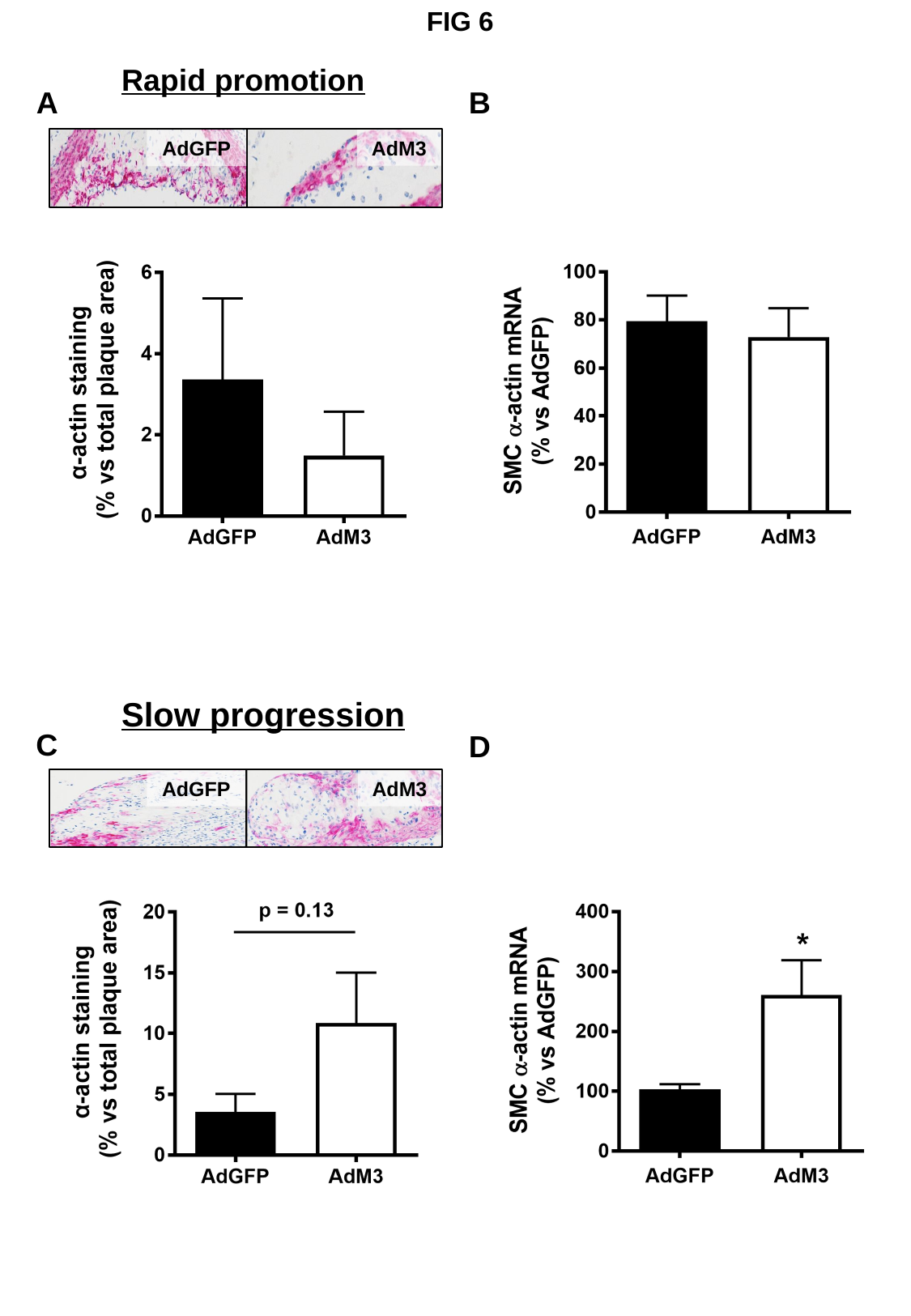

FIG 6
Rapid promotion
A
B
AdM3
AdGFP
Slow progression
C
D
AdGFP
AdM3

## Slide 4
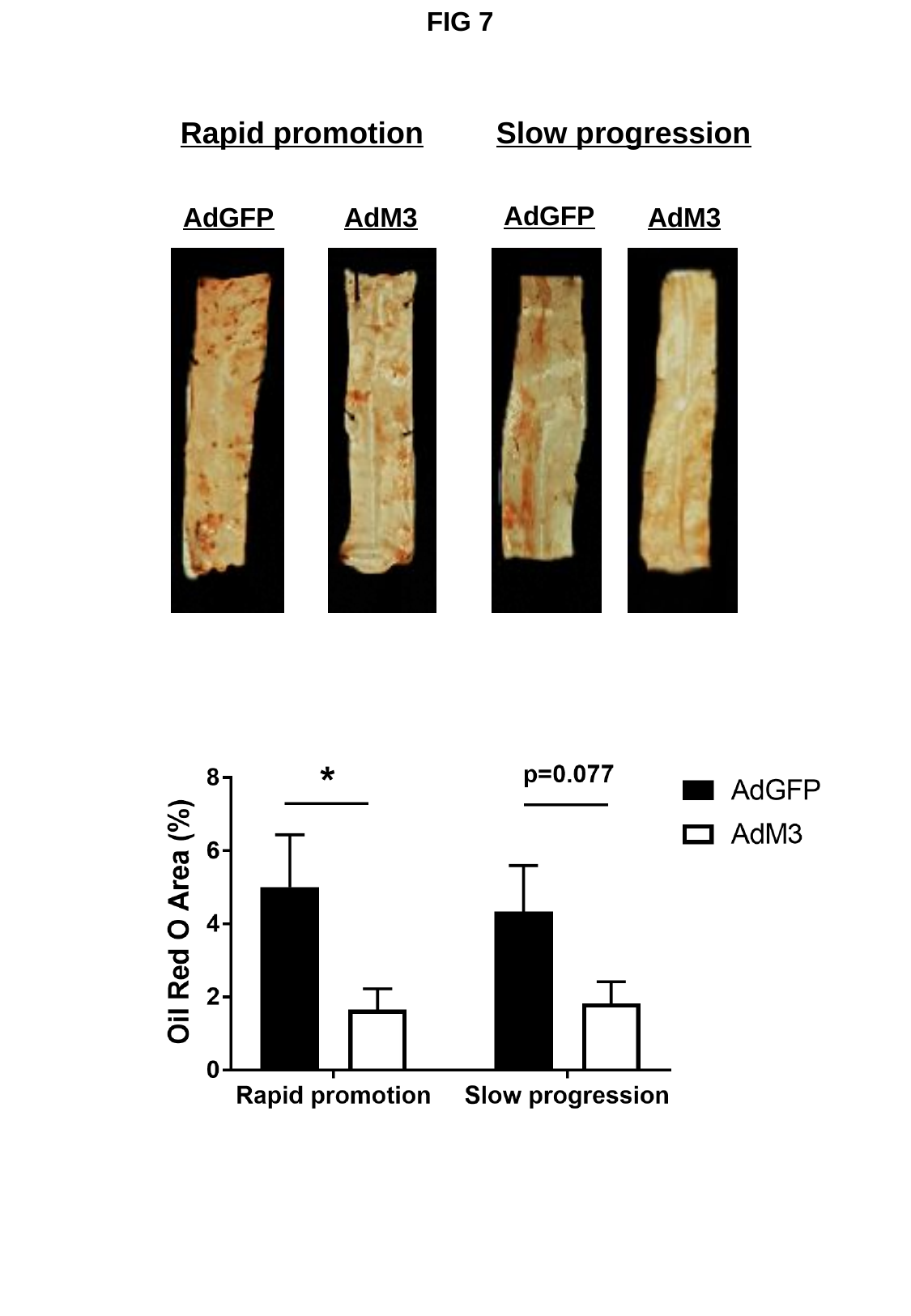

FIG 7
Slow progression
Rapid promotion
AdGFP
AdGFP
AdM3
AdM3

## Slide 5
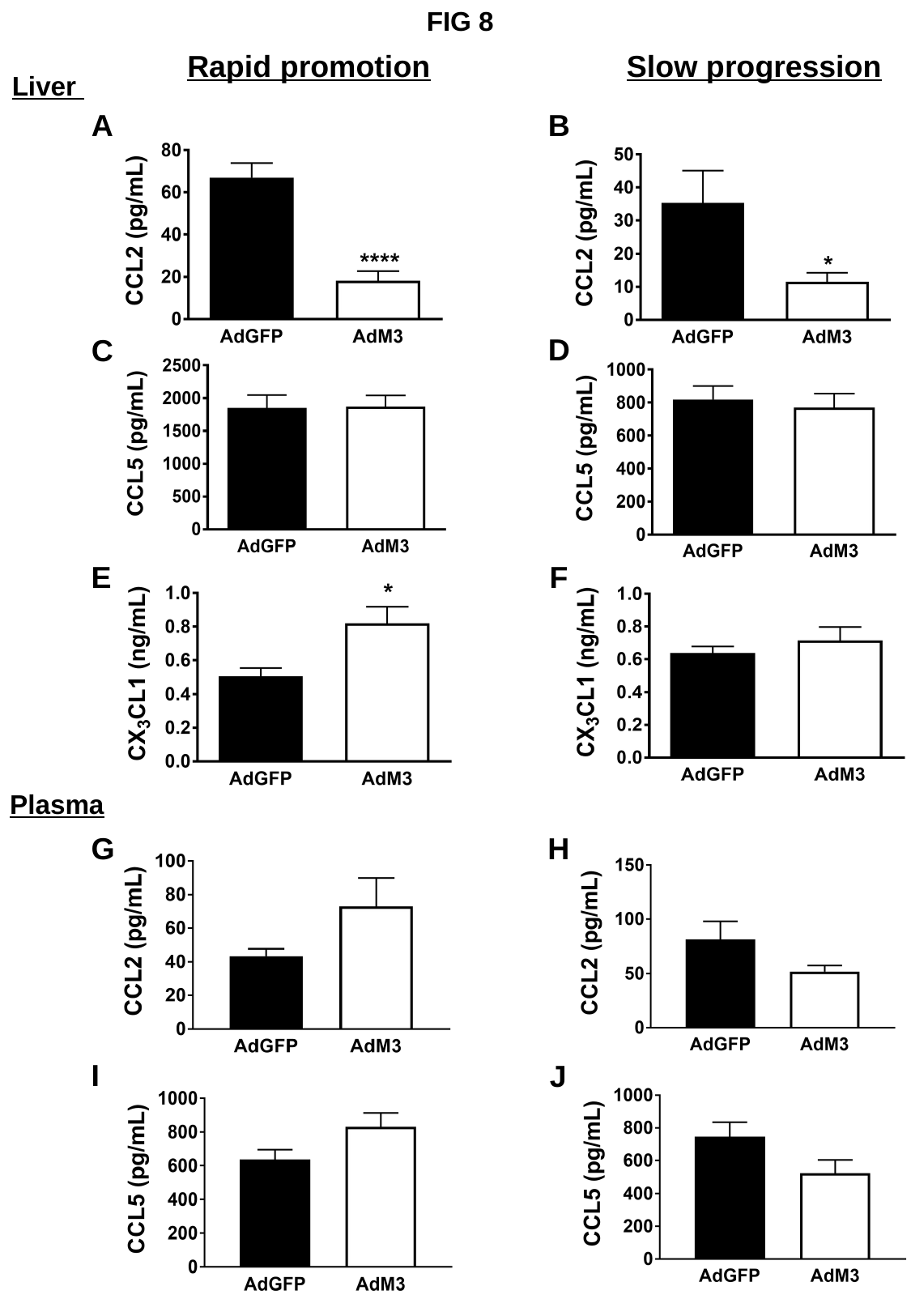

FIG 8
Rapid promotion
Slow progression
Liver
A
B
D
C
E
F
Plasma
H
G
I
J

## Slide 6
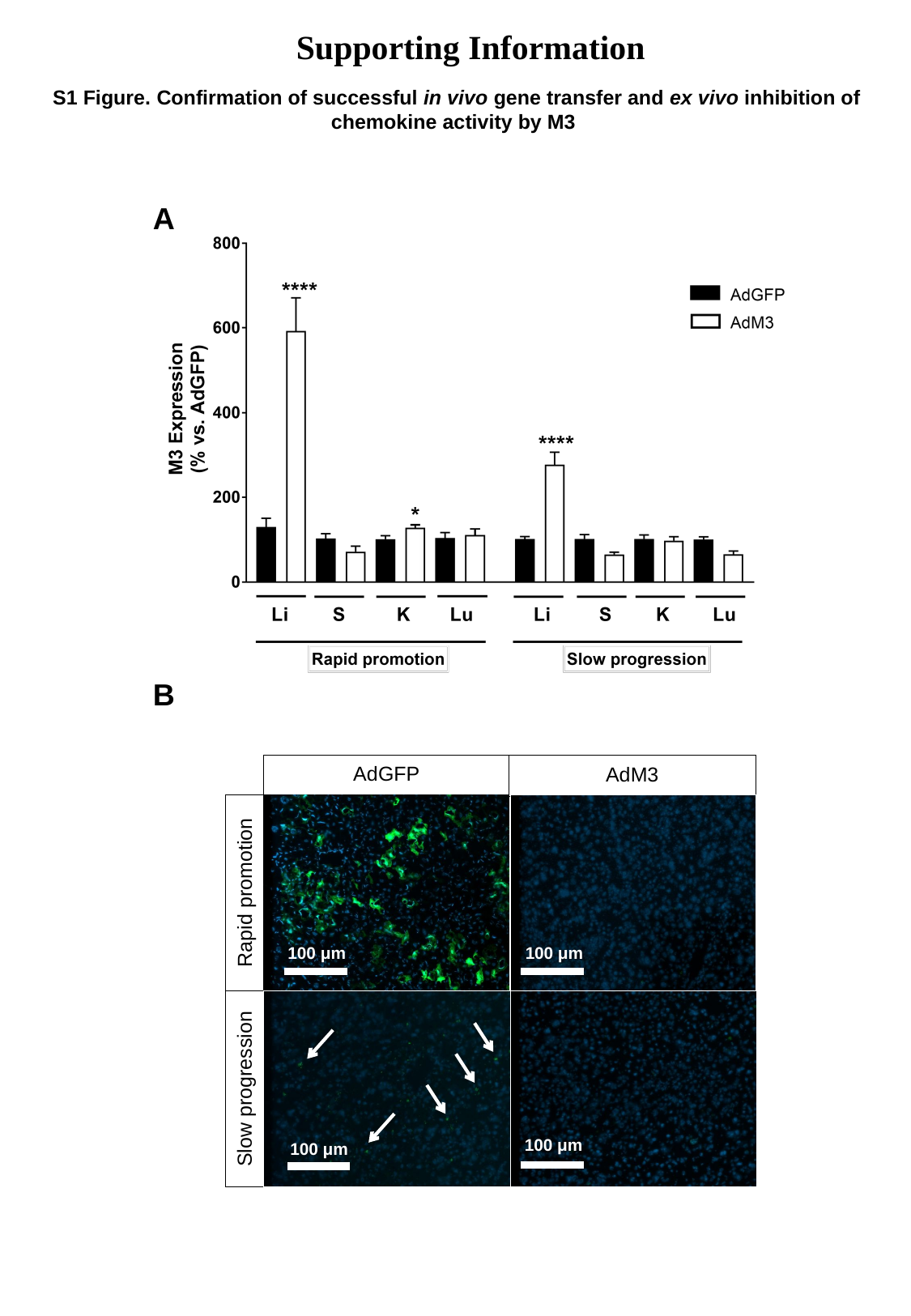

Supporting Information
S1 Figure. Confirmation of successful in vivo gene transfer and ex vivo inhibition of chemokine activity by M3
A
B
AdGFP
AdM3
Rapid promotion
100 μm
100 μm
100 μm
100 μm
Slow progression
100 μm
100 μm
100 μm
100 μm

## Slide 7
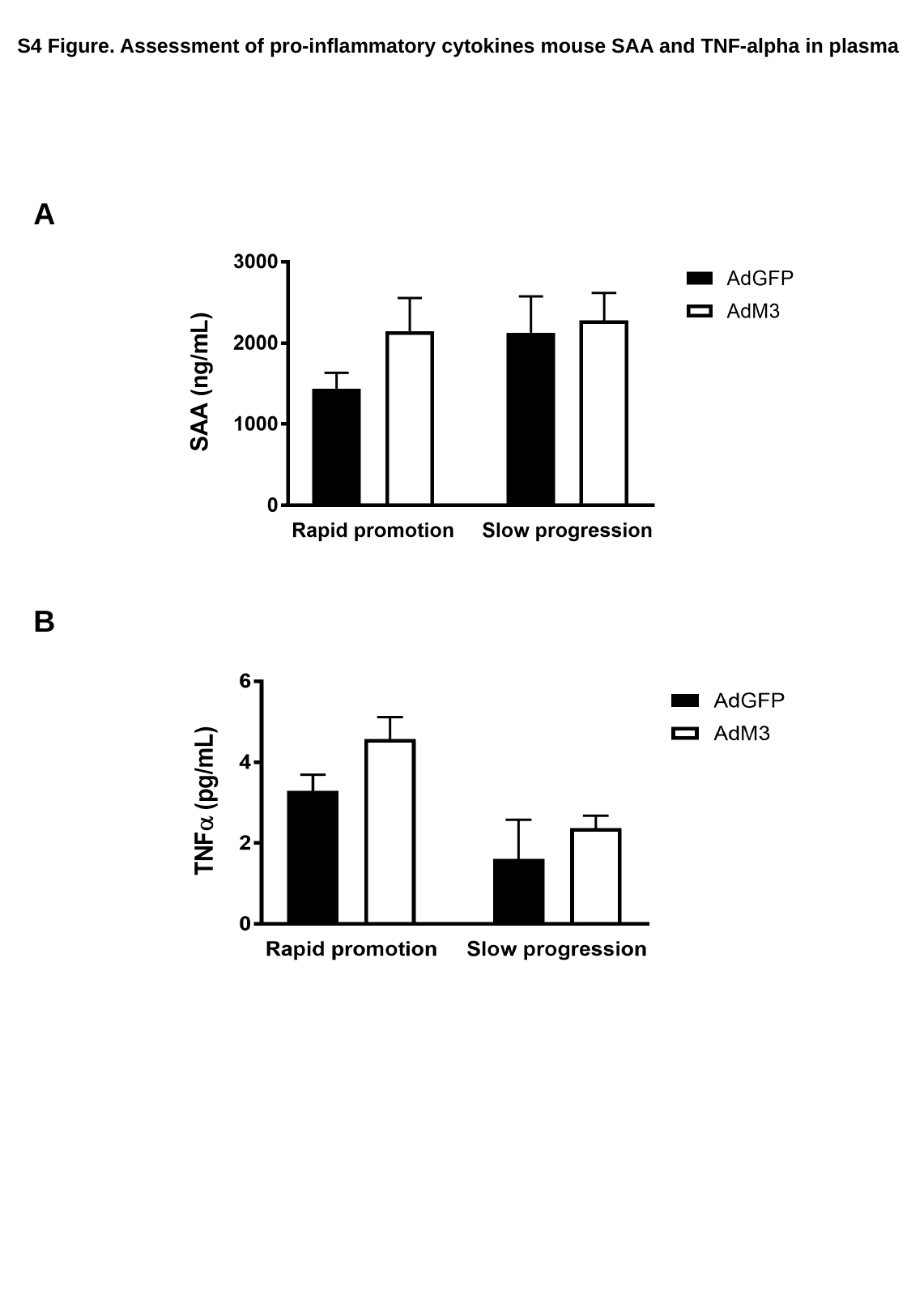

S4 Figure. Assessment of pro-inflammatory cytokines mouse SAA and TNF-alpha in plasma
A
B

## Slide 8
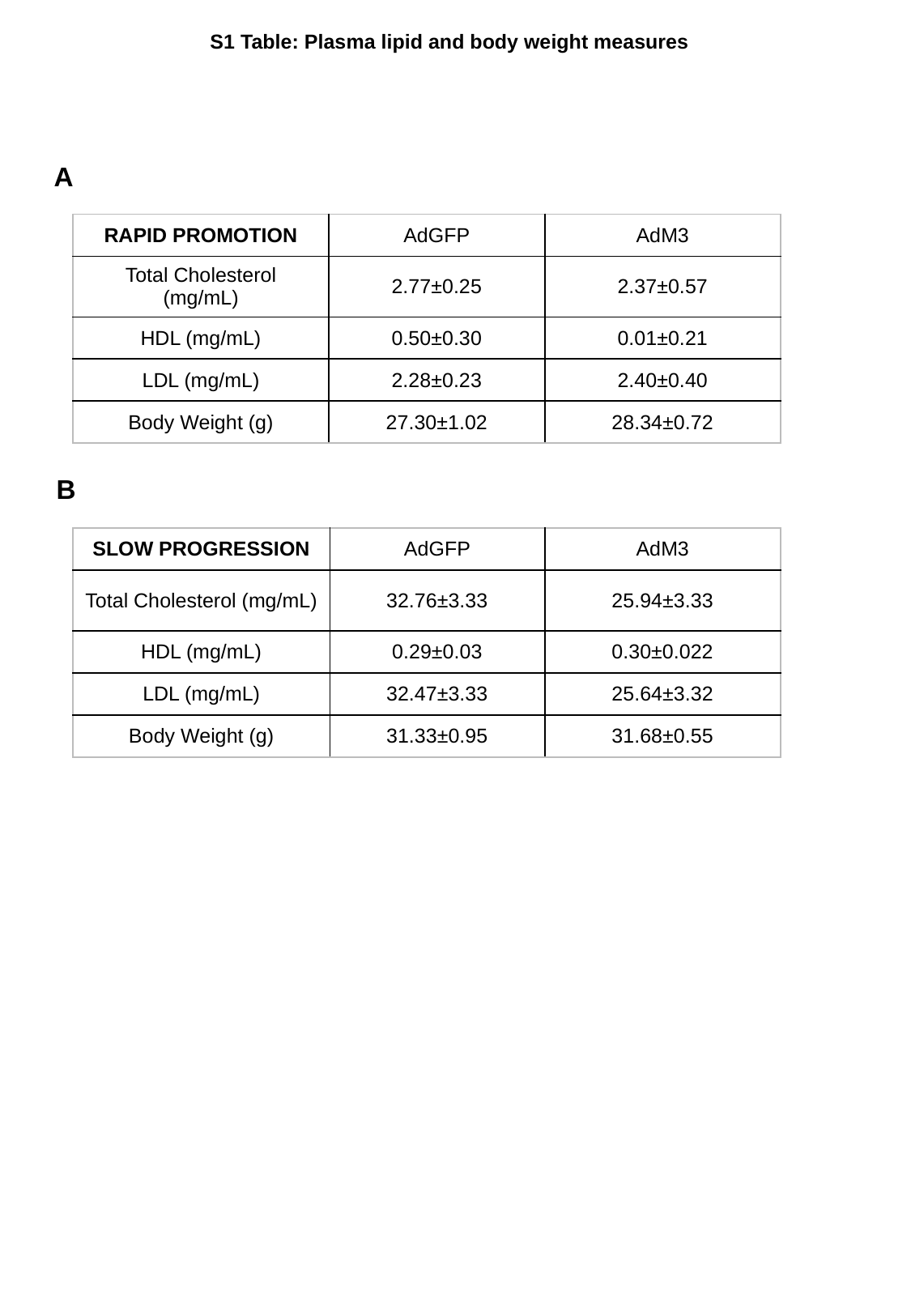

S1 Table: Plasma lipid and body weight measures
A
| RAPID PROMOTION | AdGFP | AdM3 |
| --- | --- | --- |
| Total Cholesterol (mg/mL) | 2.77±0.25 | 2.37±0.57 |
| HDL (mg/mL) | 0.50±0.30 | 0.01±0.21 |
| LDL (mg/mL) | 2.28±0.23 | 2.40±0.40 |
| Body Weight (g) | 27.30±1.02 | 28.34±0.72 |
B
| SLOW PROGRESSION | AdGFP | AdM3 |
| --- | --- | --- |
| Total Cholesterol (mg/mL) | 32.76±3.33 | 25.94±3.33 |
| HDL (mg/mL) | 0.29±0.03 | 0.30±0.022 |
| LDL (mg/mL) | 32.47±3.33 | 25.64±3.32 |
| Body Weight (g) | 31.33±0.95 | 31.68±0.55 |
